# Supplementary material for: A randomized placebo-controlled clinical trial for pharmacological activation of BCAA catabolism in patients with type 2 diabetes
Source: Nat Commun. 2022 Jun 18;13:3508. doi: 10.1038/s41467-022-31249-9 (PMC9206682; doi:10.1038/s41467-022-31249-9)
Supplement: Supplementary file 1 — Supplementary Information [file 41467_2022_31249_MOESM1_ESM.pdf]

Supplementary Information for "*A randomized placebo-controlled clinical trial for pharmacological activation of BCAA catabolism in patients with type 2 diabetes*"

SUPPLEMENTARY TABLE 1

NaPB treatment elevates muscle mitochondrial oxidative capacity [a]

	**NaPB**	**Placebo**	***P* value**
		2 weeks intervention	
State 3 MOG [b]	39.58 ± 3.39	38.30 ± 2.72	0.41
State 3 MOGS [b]	61.25 ± 4.52	57.49 ± 3.62	0.25
State u MOGS [b]	73.33 ± 6.13	71.84 ± 5.91	0.78
State 3 MPG [c]	52.27 ± 3.15	47.90 ± 3.44	0.09
State 3 MPGS [c]	74.01 ± 4.05	67.05 ± 4.05	0.04*
State u MPGS [c]	109.38 ± 7.14	99.46 ± 7.56	0.12

[a] Data expressed as mean ± SE and are pmol · mg-1 · s^{-1}. The intervention effect was analyzed using the paired student t-test. **P* values < 0.05 NaPB vs. Placebo. NaPB, sodium phenylbutyrate; MOG, malate + octanoyl carnitine + glutamate; MOGS, malate + octanoyl carnitine + glutamate + succinate; MPG, malate + pyruvate + glutamate; MPGS, malate + pyruvate + glutamate + succinate;
[b]*n* = 14, [c]*n*=13

SUPPLEMENTARY TABLE 2

NaPB treatment did not alter ectopic lipid storage [a]

	NaPB	Placebo	*P* value
IMCL [b] (%)	0.61 ± 0.09	0.50 ± 0.07	0.15
IHL (CH_2/ ($CH_{2+}H_2O$)) [c] (%)	11.11 ± 2.26	11.70 ± 2.36	0.20
PUFA [b] (%)	13.15 ± 1.48	14.70 ± 1.39	0.50
MUFA [b] (%)	42.93 ± 1.79	41.70 ± 1.62	0.51
SFA [b] (%)	43.91 ± 1.27	44.21 ± 1.67	0.88

[a] Data expressed as mean ± SE. The intervention effect was analyzed using the paired student t-test. *P values < 0.05 NaPB vs. Placebo. IMCL, intramyocellular lipid; IHL, intrahepatic lipid; PUFA, poly-unsaturated fatty acids; MUFA, mono-unsaturated fatty acids; SFA, saturated fatty acids; NaPB, sodium phenylbutyrate;

[b] n = 13, [c] n=14

SUPPLEMENTARY TABLE 3

NaPB treatment reduces plasma BCAA levels [a]

Amino acid (μmol/L)	**NaPB**	**Placebo**	***P* value**
Alanine	426 ± 16	440 ± 15	0.76
Arginine	71 ± 4	71 ± 4	0.83
Asparagine	64 ± 4	65 ± 5	0.78
Asparagine	2.2 ± 0.3	2.8 ± 0.9	0.51
Cysteine	68 ± 2	66 ± 2	0.16
Glutamic acid	122 ± 6	112 ± 7	0.17
Glycine	206 ± 12	194 ± 9	0.15
Histidine	70 ± 3	75 ± 2	0.08
Lysine	175 ± 5	183 ± 5	0.16
Methionine	23 ± 1	24 ± 1	0.07
Phenylalanine	61 ± 2	62 ± 2	0.11
Serine	89 ± 5	91 ± 4	0.63
Threonine	107 ± 5	111 ± 4	0.49
Tryptophan	49 ± 2	53 ± 2	0.01**
Tyrosine	64 ± 3	66 ± 3	0.09
Total AAA, μmol/L	174 ± 6	181 ± 6	0.004**
Total EAA, μmol/L	964 ± 18	1008 ± 24	0.004**

[a] *n*=16. Data expressed as mean ± SE. The intervention effect was analyzed using the paired student t-test. ***P* values < 0.01 NaPB vs. Placebo. Blood samples were taken after 2 weeks of NaPB treatment and placebo after an overnight fast. Total AAA includes phenylalanine, tryptophan, tyrosine; total EAA includes histidine, lysine, methionine, phenylalanine, tryptophan, tyrosine, isoleucine, leucine, valine. NaPB, sodium phenylbutyrate; AAA, aromatic amino acids; EAA, essential amino acids.

SUPPLEMENTARY TABLE 4

No change in sleeping metabolic rate and nocturnal substrate oxidation with NaPB treatment [a]

	**NaPB**	**Placebo**	***P* value**
SMR (MJ/d) [b]	7.0 ± 0.3	7.1 ± 0.3	0.92
Sleep RER [b]	0.81 ± 0.01	0.82 ± 0.01	0.58
Carbohydrate oxidation (μmol · kg^{-1} · min^{-1}) [c]	5.3 ± 0.5	5.4 ± 0.4	0.76
Fat oxidation (μmol · kg^{-1} · min^{-1}) [c]	2.2 ± 0.1	2.1 ± 0.1	0.61
Protein oxidation (μmol · kg^{-1} · min^{-1}) [c]	6.7 ± 0.5	7.0 ± 0.58	0.55

[a] Data expressed as mean ± SE. The intervention effect was analyzed using the paired student t-test. **P* values < 0.05 NaPB vs. Placebo. NaPB, sodium phenylbutyrate; SMR, sleep metabolic rate

[b]*n* = 16, [c]*n*=15

SUPPLEMENTARY Figure 1: Treatment compliance

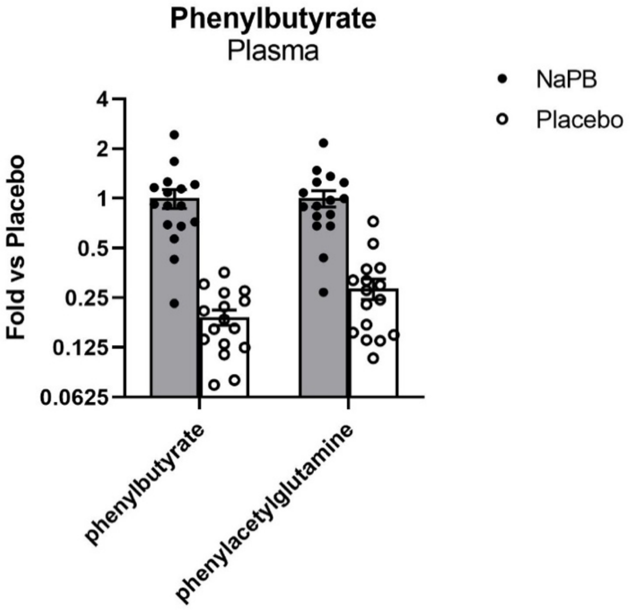

Suppl Fig. 1 Treatment compliance. Study compliance was measured in patients with T2D after 2-week treatment with NaPB (grey bars, n=16) and placebo (white bars, n=16) in plasma. Data are expressed as mean ± SE. The intervention effect was analyzed using the paired student t-test. *P < 0.05. T2D, type 2 diabetes; NaPB, sodium phenylbutyrate. Source data are provided as a Source Data file

RESEARCH PROTOCOL

Lowering Branched-Chain Amino Acids as a New Strategy to Improve Insulin Sensitivity

(April 2019)

PROTOCOL TITLE 'Lowering Branched-Chain Amino Acids as New Strategy to Improve Insulin Sensitiviy'

**Protocol ID**	**NaPB_EP**
**Short title**	**NaPB as tool to boost BCAA oxidation**
**EudraCT number**	**2018-003176-13**
**Version**	**4**
**Date**	**24-04-2019**
**Coordinating investigator/project leader**	**Froukje Vanweert<br>Universiteitssingel 50 (room 2.250)<br>6229 ER Maastricht<br>tel: 043-388 1390<br>email: f.vanweert@maastrichtuniversity.nl**
**Principal investigator(s) (in Dutch: hoofdonderzoeker/ uitvoerder)**	**Dr. Esther Phielix<br>Universiteitssingel 50 (room 0.314)<br>6229 ER Maastricht<br>tel: 043- 388 1311<br>email: esther.phielix@maastrichtuniversity.nl**
**Sponsor (in Dutch: verrichter/opdrachtgever)**	**School of Nutrition and Translational Research in Metabolism (NUTRIM)<br>Maastricht University<br>PO Box 616, 6200 MD Maastricht**
**Subsidising party**	**Diabetes Fonds Nederland (DFN)**
**Independent expert (s)**	**Prof. dr. S.W.M. Olde Damink<br>Maastricht UMC+<br>Oncologie<br>043-3881499<br>steven.oldedamink@mumc.nl**
**Laboratory sites**	**Maastricht University<br>Department of Nutrition and Movement Sciences<br>Universiteitssingel 50, 6229 ER Maastricht<br><br>Maastricht UMC+<br>Centraal Diagnostisch Laboratorium<br>PO Box 5800, 6202 AZ Maastricht<br><br>Tiofarma BV, Benjamin Franklinstraat 10, 3261 LW Oud Beijerland**

**Pharmacy**	**Apotheek Radboud Universitair Medisch Centrum Postbus 9101, 6500 HB Nijmegen**<br><br>**Apotheek Maastricht UMC+, P. Debyelaan 25 6229 HX Maastricht**<br><br>**Academisch Medisch Centrum Goederenontvangst Apotheek, t.a.v. geneesmiddelenonderzoek, Meidreef 9, 1105 AZ Amsterdam**

PROTOCOL SIGNATURE SHEET

Name	Signature	Date
Head of Department:<br>Prof. Jogchum Plat		
Coordinating Investigator/Project leader/Principal Investigator:<br>Dr. Esther Phielix		

TABLE OF CONTENTS

1. INTRODUCTION AND RATIONALE 10
2. OBJECTIVES 19
3. STUDY DESIGN 20
4. STUDY POPULATION 22
   4.1 Population (base) 22
   4.2 Inclusion criteria 22
   4.3 Exclusion criteria 22
   4.4 Sample size calculation 23
5. TREATMENT OF SUBJECTS 24
   5.1 Investigational product/treatment 24
   5.2 Use of co-intervention (if applicable) 25
   5.3 Escape medication (if applicable) 25
6. INVESTIGATIONAL PRODUCT 26
   6.1 Name and description of investigational product(s) 26
   6.2 Summary of findings from non-clinical studies 28
   6.3 Summary of findings from clinical studies 28
   6.4 Summary of known and potential risks and benefits 29
   6.5 Description and justification of route of administration and dosage 30
   6.6 Dosages, dosage modifications and method of administration 30
   6.7 Preparation and labelling of Investigational Medicinal Product 30
   6.8 Drug accountability 31
7. NON-INVESTIGATIONAL PRODUCT 32
   7.1 Name and description of non-investigational product(s) 32
   7.2 Summary of findings from non-clinical studies 32
   7.3 Summary of findings from clinical studies 32
   7.4 Summary of known and potential risks and benefits 32
   7.5 Description and justification of route of administration and dosage 32
   7.6 Dosages, dosage modifications and method of administration 33
   7.7 Drug accountability 33
8. METHODS 34
   8.1 Study parameters/endpoints 34
      8.1.1 Main study parameter/endpoint 34
      8.1.2 Secondary study parameters/endpoints (if applicable) 34
      8.1.3 Other study parameters (if applicable) 34
   8.2 Randomisation, blinding and treatment allocation 34
   8.3 Study procedures 35
   8.4 Withdrawal of individual subjects 41
      8.4.1 Specific criteria for withdrawal (if applicable) 41
   8.5 Replacement of individual subjects after withdrawal 41
   8.6 Follow-up of subjects withdrawn from treatment 41
   8.7 Premature termination of the study 41

9. SAFETY REPORTING 43
9.1 Temporary halt for reasons of subject safety....................43
9.2 AEs, SAEs and SUSARs 43
9.2.1 Adverse events (AEs)....................43
9.2.2 Serious adverse events (SAEs)....................43
9.2.3 Suspected unexpected serious adverse reactions (SUSARs)....................44
9.3 Annual safety report....................45
9.4 Follow-up of adverse events 45
9.5 [Data Safety Monitoring Board (DSMB) / Safety Committee]....................45
10. STATISTICAL ANALYSIS 46
10.1 Primary study parameter(s)....................46
10.2 Secondary study parameter(s)....................46
10.3 Other study parameters 47
10.4 Interim analysis (if applicable)....................47
11. ETHICAL CONSIDERATIONS 48
11.1 Regulation statement....................48
11.2 Recruitment and consent 48
11.3 Objection by minors or incapacitated subjects (if applicable)....................48
11.4 Benefits and risks assessment, group relatedness....................48
11.5 Compensation for injury 50
11.6 Incentives (if applicable)....................50
12. ADMINISTRATIVE ASPECTS, MONITORING AND PUBLICATION 51
12.1 Handling and storage of data and documents....................51
12.2 Monitoring and Quality Assurance....................51
12.3 Amendments....................51
12.4 Annual progress report....................52
12.5 Temporary halt and (prematurely) end of study report....................52
12.6 Public disclosure and publication policy 52
13. STRUCTURED RISK ANALYSIS 53
13.1 Potential issues of concern 53
13.2 Synthesis 56
14. REFERENCES....................57

LIST OF ABBREVIATIONS AND RELEVANT DEFINITIONS

ABR	ABR form, General Assessment and Registration form, is the application form that is required for submission to the accredited Ethics Committee (In Dutch, ABR = Algemene Beoordeling en Registratie)
AE	Adverse Event
AR	Adverse Reaction
BCAA	Branched-Chain Amino Acids
BCKD	Branched-Chain α-keto dehydrogenase
BSA	Body Surface Area
CA	Competent Authority
CCMO	Central Committee on Research Involving Human Subjects; in Dutch: Centrale Commissie Mensgebonden Onderzoek
CV	Curriculum Vitae
DSMB	Data Safety Monitoring Board
EU	European Union
EudraCT	European drug regulatory affairs Clinical Trials
GCP	Good Clinical Practice
IB	Investigator’s Brochure
IC	Informed Consent
IMP	Investigational Medicinal Product
IMPD	Investigational Medicinal Product Dossier
METC	Medical research ethics committee (MREC); in Dutch: medisch ethische toetsing commissie (METC)
Na-PB	Sodium Phenyl Butyrate
NSAID	Non-Steroidal Anti-Inflammatory Drug
OCR	Oxygen Consumption Rate
OGTT	Oral Glucose Tolerance Test
RER	Respiratory Exchange Ratio
(S)AE	(Serious) Adverse Event
SPC	Summary of Product Characteristics (in Dutch: officiële productinfomatie IB1-tekst)
Sponsor	The sponsor is the party that commissions the organisation or performance of the research, for example a pharmaceutical

	**company, academic hospital, scientific organisation or investigator. A party that provides funding for a study but does not commission it is not regarded as the sponsor, but referred to as a subsidising party.**
**SUSAR**	**Suspected Unexpected Serious Adverse Reaction**
**T2D**	**Type 2 Diabetes**
**Wbp**	**Personal Data Protection Act (in Dutch: Wet Bescherming Persoonsgevens)**
**WMO**	**Medical Research Involving Human Subjects Act (in Dutch: Wet Medisch-wetenschappelijk Onderzoek met Mensen**

SUMMARY

Rationale: Insulin resistance is the most important risk factor in Type 2 Diabetes (T2D). Several studies identified branched-chain amino acids (BCAA; leucine, isoleucine and valine) to be substantially elevated in people with T2D. Recently, I confirmed the finding of higher BCAA in people with T2D. Furthermore, I found strong associations between BCAA and key metabolic disarrangements seen in T2D at the level of mitochondrial function, liver fat, insulin resistance and metabolic flexibility. Importantly, data showed lower whole body leucine oxidation in patients with T2DM vs. control humans. Here, I want to use the FDA approved drug Pheburane containing sodium-phenylbutyrate (NaPB) **-**a drug known to lower plasma BCAA in humans via accelerated BCAA oxidation- in patients with T2DM as strategy to enhance BCAA metabolism. This project aims to investigate whether Na-PB-enhanced BCAA oxidation would be a potential strategy in people with T2D to improve metabolic health.

Objective: Primary objective is the delta change in whole body insulin sensitivity expressed as glucose disposal rate (μmol/kg/min) upon 2 weeks of Na-PB vs. placebo treatment. Secondary objectives are muscle mitochondrial oxidative capacity (pmol/mg/s), muscle and liver fat content (%) and energy metabolism (respiratory exchange ratio and kJ/kg/min).

Study design: 2-week clinical randomized controlled trial (RCT) with a double blinded, placebo-controlled, cross-over design, including a wash-out period of 6 weeks.

Study population: 18 male and (post-menopausal) female participants (50% m/ 50% f) with T2D will be randomized into the study. Participants will be relatively well-controlled (HbA1C<8.5%), are on oral glucose-lowering medication, are overweight/obese (BMI 25-38 kg/m^2) and between 45-75 years old.

Intervention: 2 weeks oral administration of 4.8 g/m^2/day sodium phenylbutyrate (in the form of Pheburane) or placebo per day. Although depending on body surface area, ~21 g Pheburane needs to be administered spread over the day in 3 times taken with a meal.

Main study parameters/endpoints: insulin sensitivity expressed as delta change in μmol/kg/min upon Na-PB treatment versus placebo. The endpoint of study is the measurement of insulin sensitivity of the 15th participant after the second intervention period.

Nature and extent of the burden and risks associated with participation, benefit and group relatedness: No direct health benefits for the participants are expected. Burdens: time investment with study visits and administration of study drug. (Low) risks of measurements: hypoglycaemia during the clamp, hematomas and inflammation upon muscle biopsies. Risks with study drug: negative nitrogen balance, loss of appetite, changed body odour (described for 3-4% of all patients using Na-PB with long administration time).

1. INTRODUCTION AND RATIONALE

Rational

Type 2 diabetes is characterized by a broad scale of metabolic disturbances. People with overt type 2 diabetes feature i.e. low mitochondrial function [1-3], are less capable in switching between fat and glucose oxidation under insulin stimulated conditions (termed as metabolic inflexibility) and are characterized by elevated fat content in liver and muscle [4]. The last decade, metabolic profiling done in several studies accentuated the presence of high systemic levels of branched-chain amino acids (BCAA) in people with type 2 diabetes [5-7]. The notion of a potential role of BCAA in obesity and diabetes, originally dates back from 1965 by Felig et al. [8]. In this report as well as in other publications [9, 10], the BCAA's were predominantly positioned as insulinogenic agents affecting (myofibrillar) protein turnover.

Nowadays, the specific elevated cluster of BCAA's (isoleucine, leucine and valine) in the circulation is thought to be a diagnostic marker in identifying people at risk to develop diabetes [6, 11] based on the strong positive association with insulin resistance, the main risk factor in the development of type 2 diabetes [6, 11-16]. In addition, reports indicate that humans are characterized by elevated BCAA prior to the actual onset of type 2 diabetes [5, 7], highlighting that BCAA's could function as well as prognostic biomarker for the development of the disease. It is however unknown why BCAA's are elevated in insulin resistant people with or without diabetes and whether BCAA's play a role in the pathophysiology of diabetes.

For long time, elevated systemic BCAA's in patients with type 2 diabetes were supposed to be a consequence of the insulin resistant state, due to blunted insulin-suppressed protein breakdown in muscle [17]. The insensitivity of the BCAA catabolic pathway towards the suppressive action of insulin together with a nutrient overload are indeed potential factors underlying systemic elevation of BCAA's. An alternative hypothesis is a diminished oxidation of BCAA in type 2 people with diabetes. In line, a genetic Mendelian Randomisation study reported a significant locus involved in BCAA metabolism located at the phosphatase gene, which correlated with the incidence of diabetes [18]. This finding point toward a causal role of disturbed BCAA metabolism involved in the pathophysiology of diabetes. However, two other Mendelian Randomisation studies showed opposite directions of causality [7, 19]. With this, it remains inconclusive whether diminished BCAA oxidation is causal in the development of type 2 diabetes or not.

With a recently finished study we aimed to obtain more insight into the role of BCAA metabolism in people featuring type 2 diabetes and people (paper in preparation). First we

explored whether BCAA were elevated in both older and overweight patients with type 2 diabetes and in people at high risk for the development of the disease (first-degree relatieves; FDR) compared to a control group. We furthermore explored if systemic circulating BCAA's did associate with key metabolic disturbances seen in type 2 diabetes, like insulin sensitivity, mitochondrial function, metabolic flexibility and hepatic fat content.

We concluded that patients with overt type 2 diabetes have high systemic BCAA levels corresponding to low whole body BCAA oxidation rates. Low BCAA oxidation would result in reduced mitochondrial function, and associate with enhanced ectopic lipid storage and insulin resistance. Our goal is to investigate this hypothesis in humans, as BCAA metabolism has been reported to be significantly different from rodents [20], although therewith facing restrictions i.e. collecting human tissue. Recently we explored the associations between circulating BCAA's and key metabolic parameters in patients with and without type 2 diabetes (i.e. intrahepatic fat content, mitochondrial function and metabolic flexibility). Also, we measured whole body BCAA oxidation rates in patients with type 2 diabetes and control participants, to explore eventual differences in BCAA catabolism. Based on results obtained, here we boost the BCAA oxidation using Pheburane to see if metabolic aberrations in T2DM will improve.

Diminshed BCAA oxidation would impact is important in orchestrating oxidative competition at mitochondrial level between substrates, both in liver as in skeletal muscle. This could possibly influence the physiological insulin resistant state. The oxidation of BCAA involves the rate-limiting branched-chain alpha-ketoacid dehydrogenase (BCKD) complex, regulated by a phosphorylating kinase (thereby inactivating BCKD activity) and a dephosphorylating phosphatase (thereby activating BCKD activity). More over, BCAA catabolism drives the production of propionyl-CoA (precursor of acetyl-CoA) and succinyl-COA, pivotal intermediates of the tricarboxylic acid (TCA) cycle. As these TCA cyclus intermediates are crucial in anaplerotic reactions, BCAA metabolism influences mitochondrial capacity. Basic knowledge on anaplerotic reactions origin from 1960 [21]. As BCAA oxidation gives rise to TCA intermediates, altered availability of these BCAA-derived intermediates could therefore affect mitochondrial fuel flexibility and possibly the physiological insulin resistant state [22, 23].

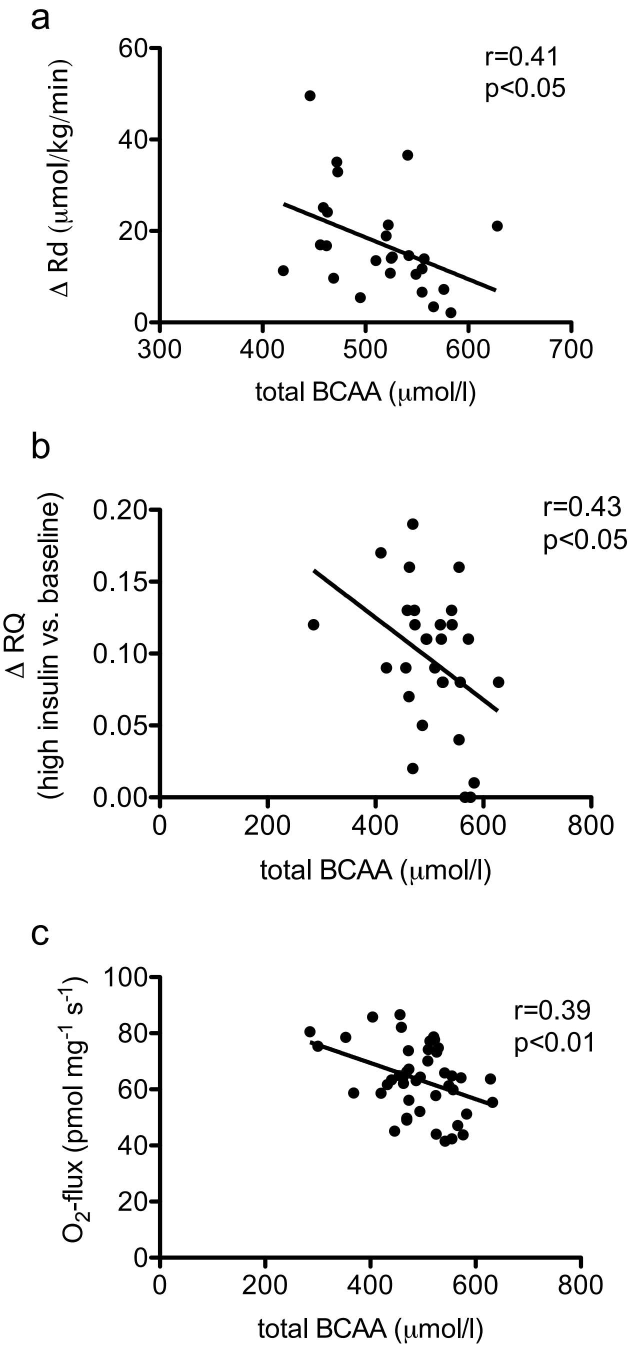

***Figure 1**: associations between systemic BCAA and insulin sensitivity (a), metabolic flexibility (b) and muscle mitochondrial oxidative capacity (c) in people with T2D, first-degree relatives of people with T2D and in healthy control participants. Results obtained from a recently finished observational study in which I investigated the associations between BCAA and metabolic read-out parameters.*

Pre-clinical studies (humans)

Na-PB has been successfully applied as a BCAA lowering agent in patients with MSUD or urea cycle disorder, but also in healthy humans possessing normal BCAA levels before Na-PB was administered. More specifically, in healthy people Na-PB induced a drop in leucine ranging from 26-51%, a drop in isoleucine between 33-50% and a drop in valine round 30% [24-28]. It is of relevance to note that the mode of action of Na-PB is via activation of the BCKD complex. In T2D, the activity of this enzyme in skeletal muscle is reported to be compromised by ~63%

[20] leaving ample room for improvement. Thus, the currently available data does not provide reasons to believe that the BCAA lowering capacity of Na-BP would be different in patients with T2D (a population who have higher BCAA levels to start with).

In humans, 50-60% of the oxidative capacity of BCKD resides within skeletal muscle. These data originate from a study that used molecular probes to measure the BCAA-catabolism by measuring the activity of BCKD and systematically compare the activity of a variety of tissues in man and rats [20]. Moreover, it is of relevance to note that 75-80% of post-prandial glucose uptake in humans resides within skeletal muscle [29]. Hence, also modest improvements in BCKD activity in skeletal muscle percentage wise may have profound effects on whole body glucose uptake. I would also like to show here my latest ‘proof-of-concept’ pilot data obtained in cultured human myotubes (**figure 2**), indicating that (in line with my hypothesis) muscle mitochondrial function improved upon administration of Na-PB to the petri-dish.

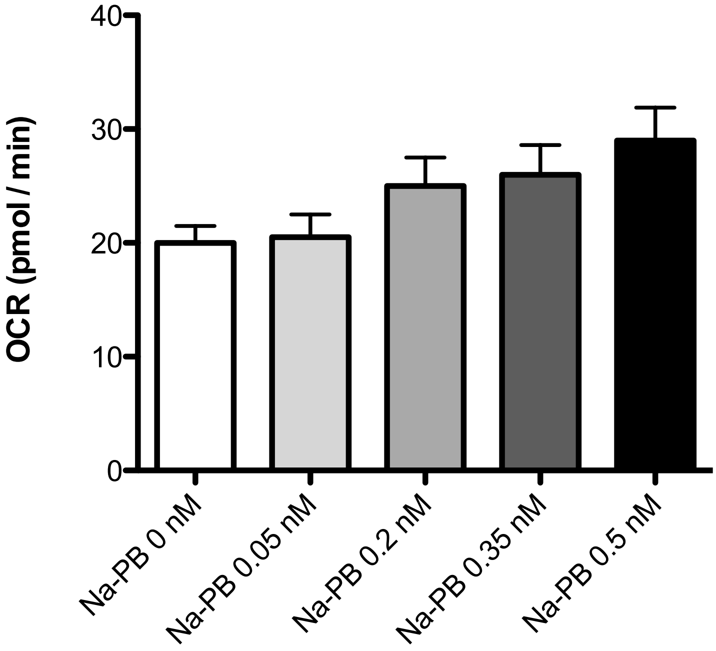

Figure 2: *The effect of increasing concentrations of 24 hours incubation of Na-PB on mitochondrial function (oxygen consumption rate, OCR) measured in cultured human myotubes obtained from patients with T2D in a Seahorse system.*

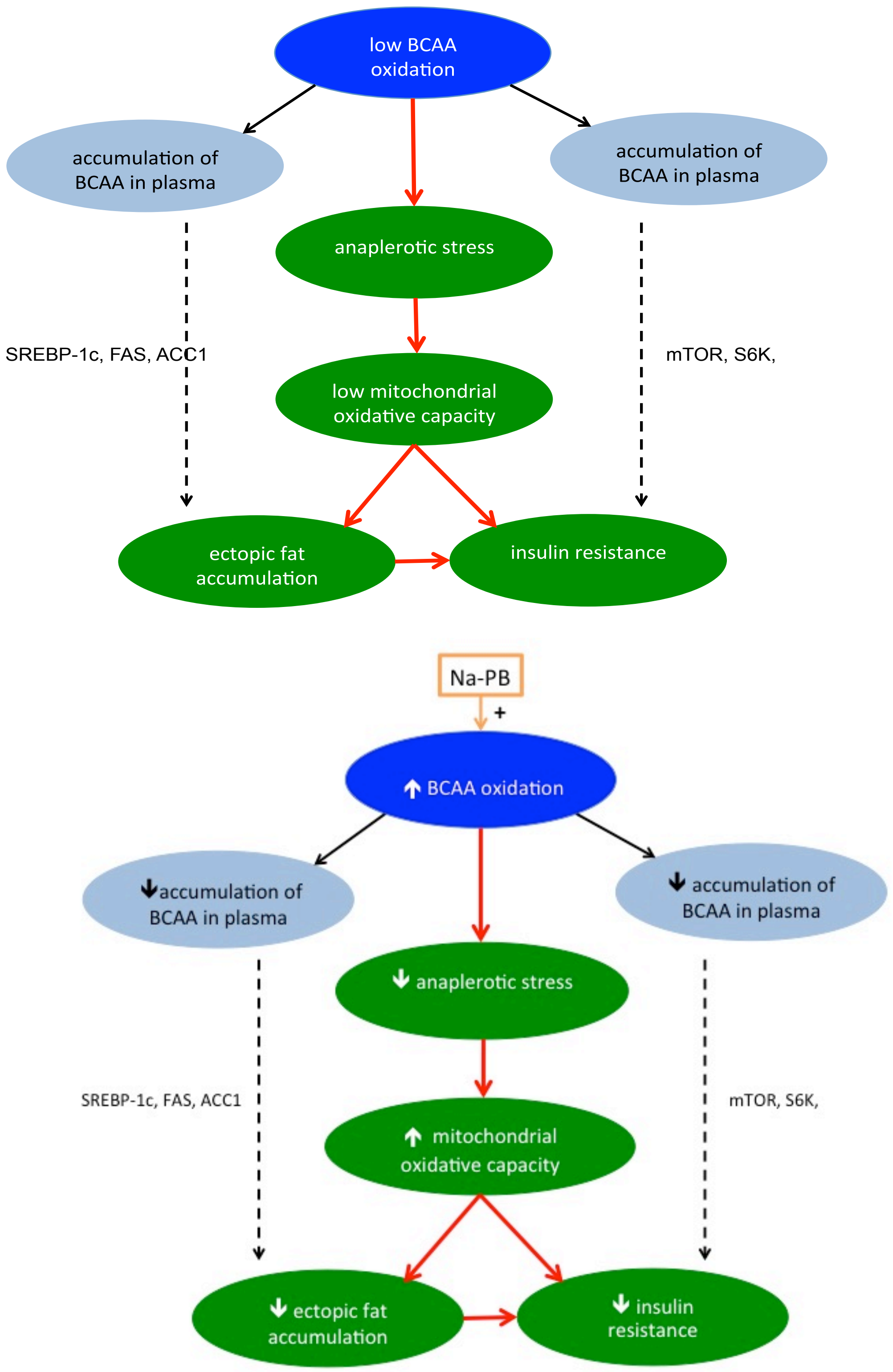

***Figure 3**: A schematic overview of the general working hypothesis (top panel). Major well-known metabolic parameters involved in the development of insulin resistance and T2D, including low mitochondrial function and elevated fat content in muscle and liver. In the lowest panel the hypothesized effects are shown for Na-PB.*

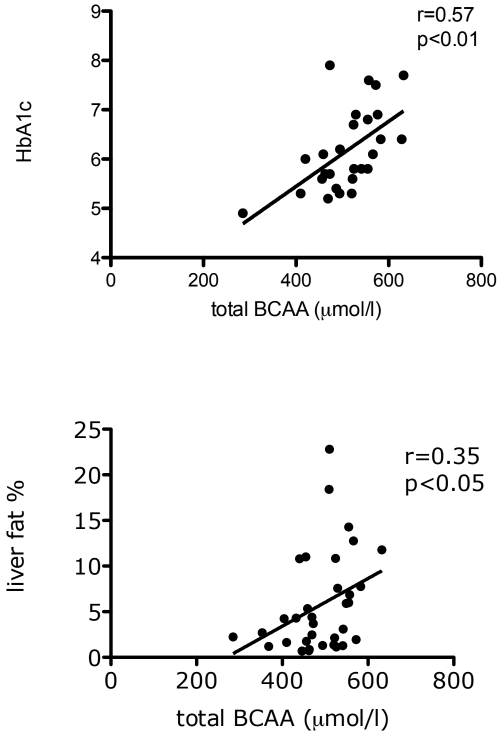

Figure 4: *Strong positive associations found in patients with T2D, obese humans without T2D and people at risk to develop T2D (first degree-relatives) between circulatory BCAA and HbA1c (glycemic control) and liver fat content.*

Hypothesis

The top panel of **figure 3** shows my general working hypothesis. In the following paragraphs I will explain this hypothesis and the rational behind. The lower panel of **figure 3** shows the expected effects of Na-PB on the general working hypothesis.

BCAA oxidation

As previously mentioned, I recently finalized a study in which leucine oxidation values (leucine as one of the BCAA) were determined in a small group of people with T2D and in healthy, control participants. Participants underwent a 2-step hyperinsulinemic clamp (including a basal phase, low-insulin and high-insulin infusion steps) paralleled by a co- infusion of 1-^{13}C leucine isotopic tracer. Leucine oxidation values were calculated based on $^{13}CO_2$ enriched breath samples and alpha-ketoisocaproaic acid in plasma. These promising pilot data indeed show that people with overt T2D are characterized by compromised whole body leucine oxidation levels during fasting as well as under insulin-stimulated conditions compared to control participants with similar age and BMI. *I generally hypothesize that low oxidation of BCAA in*

people with T2D underlies the systemically elevated concentrations. Na-PB is known to enhance BCAA oxidation via its interaction with the BCKD complex. Therefore, *here I hypothesize that Na-PB administration in patients with T2D would lower BCAA plasma concentrations via accelerated oxidation*.

BCAA and insulin sensitivity

Elevated levels of a mixture of amino acids were previously reported to directly impede with the insulin-stimulated glucose uptake in human muscle, causing insulin resistance via activation of signalling via mTOR, mTORC1 and S6K [30, 31]. *I generally hypothesize that elevated circulating BCAA could activate mTOR, mTORC1 and S6K activation thereby reducing insulin sensitivity.* Na-PB is known to lower BCAA via its interaction with the BCKD complex. Therefore, *here I hypothesize that Na-PB administration in patients with T2D would improve whole body insulin sensitivity*.

BCAA and mitochondrial function

Succinate-CoA and acetyl-CoA are two of the end product of BCAA oxidation (**figure 5**). These intermediates are part of the TCA cycle and therefore are potential in the oxidation rate of glucose and fatty acids, depending on the TCA cycling capacity inside the mitochondria. In my previous observational study I found strong negative associations between circulating BCAA and mitochondrial function as shown in **figure 1c**. *My general hypotheses state that low availability of the BCAA-derived intermediates due to low BCAA oxidation underlies low mitochondrial function in patients with T2D (**figure 5**).* Na-PB is known to enhance BCAA oxidation via its interaction with the BCKD complex. Therefore, it could be anticipated that with enhanced BCAA oxidation more TCA intermediates will be formed resulting in better TCA cycling. *Here I hypothesize that Na-PB administration in patients with T2D would improve muscle mitochondrial function*.

BCAA and energy metabolism

Improved mitochondrial function and insulin sensitivity is associated with better fuel switch regulation -in other words- better metabolic flexibility [32]. In my previous observational study I found strong negative association between circulatory BCAA and metabolic flexibility expressed as the delta respiratory exchange ratio (RER). Whether altered mitochondrial function (measured ex vivo) upon the described intervention strategy will be reflected by improved resting and sleeping energy expenditure (both measured in vivo), should be evaluated. Resting and sleeping energy expenditure are important determinants contributing to our total daily energy expenditure. *I hypothesize that administration of Na-PB in patients*

with T2D would accelerate BCAA oxidation resulting in improved resting and sleeping energy expenditure.

BCAA and ectopic fat accumulation

Together with my observation of the strong relation between BCAA and in vivo values for liver fat content (**figure 4**), this supports the notion that BCAA are involved in fat depositioning in the liver. In line, some studies performed in animals indeed showed that BCAA could stimulate ectopic fat accumulation via activation of lipogenic genes [33, 34] as depicted in **figure 3** as well. Therefore, *I generally hypothesize that BCAA stimulates expression of lipogenic genes causing increased fat accumulation in muscle and liver*. Circulatory BCAA concentrations will decrease upon the administration of Na-PB. Therefore, *here I hypothesize lower fat accumulation in liver and muscle upon administration of Na-PB in patients with T2D*.

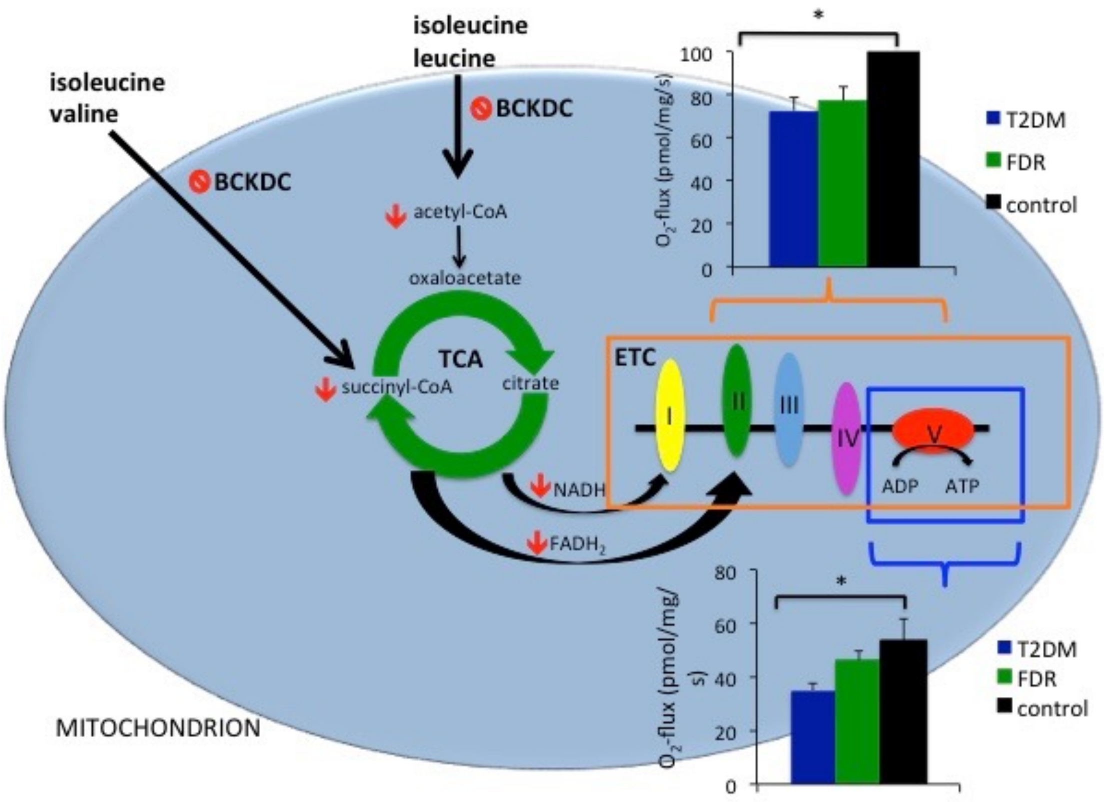

Figure 5: *the relation between the postulated diminished BCAA catabolism via lowered BCKDC activity and mitochondrial function in patients with T2D. Total electron transport chain (ETC) functioning (depicted in the upper graph) and oxidative phosphorylation (depicted in the lower graph) are lower in patients with T2D (adapted from Phielix et al. [35]). A reduced BCKDC activity possibly is the downstream underlying factor for lower mitochondrial function in T2D.*

Scientific and social relevance

The long-term aim of my research line is to define a novel therapy in T2D with a focus on new key metabolic disturbances. My goal is to explore a new strategy thereby exerting sustained improvements of metabolic health in patients with T2D, to slow down the progression of the disease, ultimately to create a beneficial clinical outcome. The research hypothesis outlined in in the previous section is new and aims at the pivotal role of amino acid metabolism in T2D. Till now, much diabetes research focuses solely on glucose and lipid metabolism, with the ignorance of amino acids as potential contributor to the metabolic disturbances seen. Here, I described however the scientific rational for investigating amino acid metabolism in T2D and provided supporting pilot data to strengthen my hypothesis. With this clinical intervention study I am very much motivated to further explore the role of BCAA metabolism in T2D to 1) gain more knowledge on the impact of BCAA metabolism on well-known metabolic impairments and 2) to explore new treatment strategies. More over, the treatment strategy described here aims to hit several key metabolic derangements seen in T2D. The surge for a treatment

strategy aiming at several metabolic derangements at the same time should be encouraged, as this would increase the change for the actual improvement of metabolic health in T2D. With this in mind, Na-PB administration as tool to elevate BCAA oxidation should be further explored ultimately to come to a potential add-on therapy to treat T2D.

The social value of this research is the ultimate improvement in health. Here I propose a translational research project with clinical instrumental value as it generates knowledge. This knowledge potentially contributes to the improvement in health. These improvements would ultimately constitute the social value of the performed research. The risks or intervention the participants will undergo in this project are justified in relation to the expected benefits to society, with providing generalizable knowledge. So, I belief my translational research including the exploration of a potential new intervention therapy out outlined here, has a great anticipated social value ultimately improving health of patients with diabetes.

2. OBJECTIVES

Primary Objective:

- Does treatment of sodium phenylbutyrate improve whole-body insulin sensitivity in patients with T2D?

Secondary Objective(s):

- Does treatment of patients with T2D with sodium phenylbutyrate improve muscle mitochondrial function?
- Does enhanced mitochondrial function precede the improvement of insulin sensitivity during the treatment with sodium phenylbutyrate in patients with T2D?
- Does treatment of patients with T2D with sodium phenylbutyrate enhance whole-body energy metabolism?
- Does treatment of patients with T2D with sodium phenylbutyrate decrease fat accumulation in muscle and the liver?

3. STUDY DESIGN

In total, 18 patients with T2D will be randomized to a double-blind, placebo-controlled, cross-over RCT. The study ends when 15 participants completely ended the second intervention period. Participants will be daily administered with ~21 g of Pheburane (4.8 g NaPB/m^2 /day) or placebo for 14 days (**figure 6**).

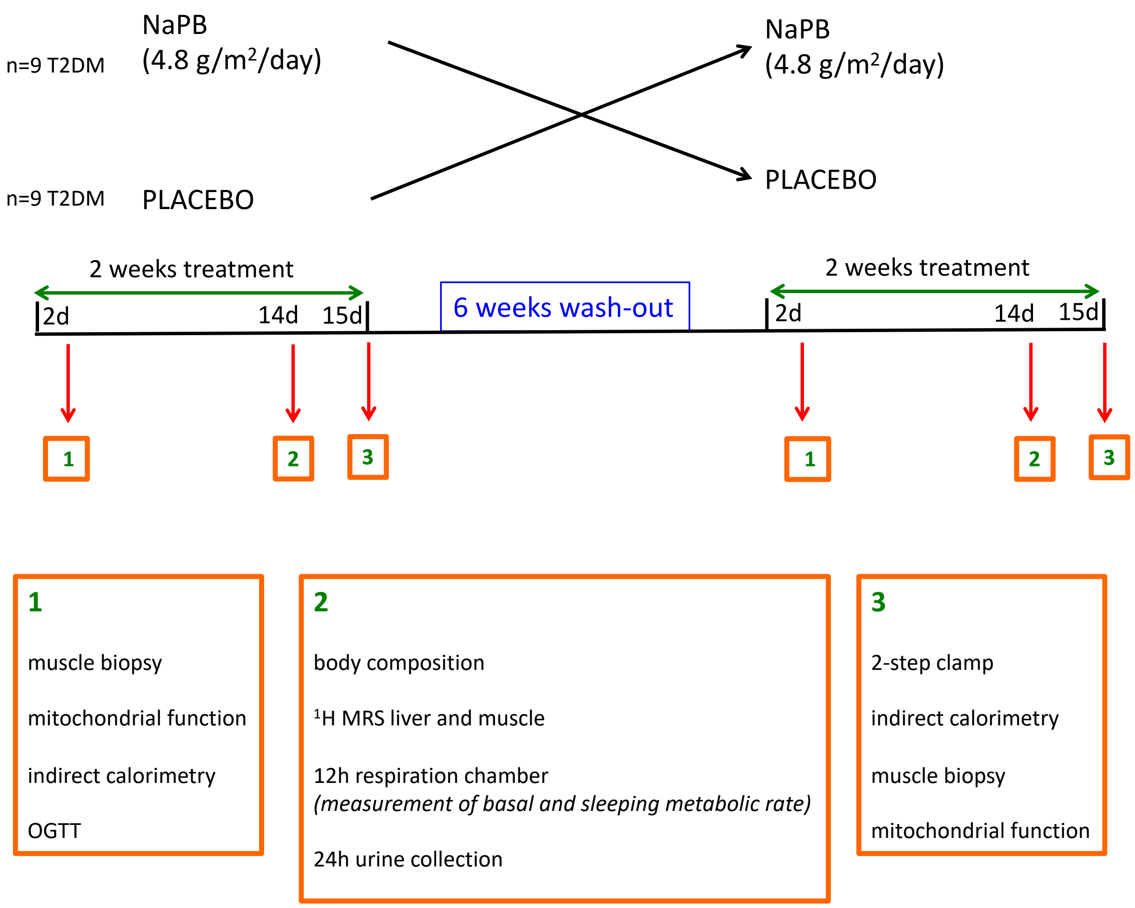

Figure 6: *double-blind, placebo-controlled, cross-over RCT in which 18 participants with T2D will be randomized to the study. All participants will be allocated in a randomised manner. Several metabolic read-out parameters will be evaluated upon treatment. From day 14 on participants will stay 12 hours in the metabolic chambers (respiration chambers) to evaluate basal and sleeping metabolic rate and collect their urine for 24 hours to calculate nitrogen balance. This stay will ensure standardized settings, like food intake and physical activity, before undergoing the 2-step clamp performed at day 15. Measurements within the first treatment period will be repeated within the second treatment period. A wash-out of 6 weeks will be maintained.*

<u>Rational study design</u>

Our main goal is to investigate whether a prolonged enhanced BCAA catabolism would improve insulin sensitivity in patients with T2D, which is the main risk factor for the development of the disease. The rational for this is based on preliminary results pointing towards diminished BCAA catabolism in patients with T2D. Based at our preliminary results in primary human myocytes, we hypothesize that sodium phenylbutyrate enhance mitochondrial function. We expect that enhanced mitochondrial function in T2D would precede improved insulin sensitivity. To understand the working mechanism of sodium phenylbutyrate, it is important to evaluate mitochondrial function (hypothesized to be the driving factor for improving insulin sensitivity), as well as whole-body energy metabolism and indirect markers of insulin sensitivity in an early stage of prolonged treatment. With the collection of muscle tissue, performance of an OGTT and indirect calorimetry upon 2 days, we are able to evaluate the working mechanism of sodium phenylbutyrate to understand outcomes obtained upon 2 weeks of treatment.

With a wash-out period of 6 weeks and the well-known pharmacokinetics of the active substance of Pheburane (Na-PB) (half-life after administration of 5 grams 0.77 hours) and half-life of 1.15 hours for phenylacetate (the ultimate active substance of the drug), there is little reason to think that a 6-week wash-out would not suffice. The direct intended effect of Na-PB is a decrease of circulating BCAA, hypothesized leading to improved insulin sensitivity (our main readout parameter). For all anti-diabetic medication currently on the market, positive effects on glucose homeostasis have already disappeared within days upon withdrawal of the medication. For physical training (which is seen as one of the most potent ways to improve insulin sensitivity), positive effects also disappear completely within 3-4 weeks after the last training session. Therefore in my opinion it is unlikely that there is still a chance of a carry-over effect after 6 weeks of washout. Furthermore, it is good to realize that by means of randomization of the drug and the placebo these possible carry-over effects are limited.

The strength of a cross-over design lies in the fact that the patients are their own controls, so that spread within the (already heterogeneous) group of patients with T2D, has only a limited effect on the statistical analysis of the possible intervention effect. In a cross-over trial, the number of subjects that need to be measured can therefore be lower than in a parallel design. Due to the complex and invasive nature of our detailed measurements, we can only measure a limited number of people within the course duration of this project. This also contributes to our preference for a cross-over design.

4. STUDY POPULATION

4.1 Population (base)

The study population will consist of 18 Caucasian males and females with diagnosed T2D who have been on stable dose of oral glucose lowering medication only for at least the last 3 months or are drug naive. Age range will be between 40 and 75 years old and BMI within 25 and 38 kg/m2. Patients must be able to provide written informed consent, meet all the inclusion criteria and none of the exclusion criteria.

4.2 Inclusion criteria

In order to be eligible to participate in this study, a subject must meet all of the following criteria:

1. Patients are able to provide signed and dated written informed consent prior to any study specific procedures
2. Women are post-menopausal (defined as at least 1 year post cessation of menses) and aged ≥ 45 and ≤ 75 years. Males are aged ≥ 40 years and ≤ 75 years
3. Patients should have suitable veins for cannulation or repeated venipuncture
4. Caucasians
5. BMI: 25-38 kg/m^2
6. Diagnosed with T2D at least 1.5 years before the start of the study
7. Relatively well-controlled T2D: HbA1c < 8.5%
8. Oral glucose lowering medication: metformin only or in combination with sulfonylurea agents and/or on stable dose of a DPPIV inhibitor treatment for at least the last 3 months
9. No signs of active diabetes-related co-morbidities like active cardiovascular diseases, active diabetic foot, polyneuropathy or retinopathy
10. No signs of active liver or kidney malfunction

4.3 Exclusion criteria

A potential subject who meets any of the following criteria will be excluded from participation in this study:

1. Previous enrolment in a clinical study with an investigational product during the last 3 months or as judged by the Investigator
2. Participate in physical activity more than 3 times a week
3. Unstable body weight (weight gain or loss > 5 kg in the last three months)
4. Insulin dependent T2D
5. Patients with congestive heart failure and and/or severe renal and or liver

insufficiency or known sodium retention with oedema

6. Patients using Probalan (probenecid), Haldol (haloperidol), Depakene (valproate) or medical products containing corticosteroids
7. Men: Hb <8.4 mmol/L, Women: Hb <7.8 mmol/l
8. Any contra-indication MRI scanning. These contra-indications include patients with e.g. the following:
   - Central nervous system aneurysm clip
   - Implanted neural stimulator
   - Implanted cardiac pacemaker of defibrillator
   - Cochlear implant
   - Metal containing corpora aliena in the eye or brains

Volunteers will be screened for contraindications with the contraindication list (see document F1.2)

A medical doctor will judge participation eligibility based on the medical history questionnaire, medication use and fasting blood parameters. If the medical doctor advises that a patient cannot participate, the patient will be excluded from enrollment.

When participants are eligible to participate in the study, the GP and the pharmacist of the participant will be contacted to check medication use.

4.4 Sample size calculation

The primary endpoint is insulin sensitivity. Insulin sensitivity is expressed as the glucose disposal rate (GDR) (umol/kg/min), which we expect to be higher in the Na-PB treatment arm. Previous cross-over intervention studies in people with T2D performed by our research group found the between person variation of GDR to be ~3.53 µmol/kg/min with a mean GDR of 35 µmol/kg/min. So far, no studies have been performed that address the effect of Na-PB treatment on insulin sensitivity in people with T2D. Therefore, we expect the mean difference is ~ 9,5 % , based on other intervention studies addressing insulin sensitivity in people with T2D [36, 37]. The effect size (Cohen's d) is calculated to be 0.93 based on the SD of difference (3.53 µmol/kg/min) and the mean of difference ((35 µmol/kg/min * 1.095) – 35 µmol/kg/min = 3.3 µmol/kg/min) (calculated using G*Power) a two-sided alpha of 0.05, and a power of 0.90, a minimal of 15 participants needs complete the study (calculated using G*Power 3.1 software, Faul, Erdfelder, Land and Buchner, University of Trier). Accounting for a possible drop-out rate of 20%, a total of (15 x 1.20=18) 18 subjects will be randomized into the study. Screen failure rate has been estimated to be approximately 30-40% and therefore we expect to include approximately 23-25 patients.

5. TREATMENT OF SUBJECTS

5.1 Investigational product/treatment

Sodium-phenylbutyrate (Na-PB)

Pheburane with Na-PB as active substance, is a FDA-approved medical prescribed drug for the management of urea cycle disorders. Na-PB helps the body to get rid of excess nitrogen waste by bypassing the urea cycle. Eating protein brings nitrogen into the body, which is then transformed into ammonia. Patients with urea-cycle disorders cannot get rid of ammonia from the body, ultimately resulting in health problems. Na-PB is administered in the form of the medication with brand name ‘Pheburane’. Pheburane are special granules taken with drinks or solid food spread over the day. The active substance in Pheburane, sodium phenylbutyrate, is converted into a substance called phenylacetate in the body. Phenylacetate combines with the amino acid glutamine, which contains nitrogen, to form a substance that can be removed from the body by the kidneys. This allows the levels of nitrogen in the body to decrease, reducing the amount of ammonia produced.

Interestingly, Na-PB effectively suppresses circulating BCAA via elevated mitochondrial **branched-chain alpha-keto acid dehydrogenase** (BCKD) complex activity thereby enhancing BCAA oxidation [38]. Na-PB has been given to several populations, ranging from healthy people to humans suffering from diseases, which are diseases different from urea cycle disorders. In these populations circulatory concentrations of BCAA decreased due to Na-PB-stimulated BCAA oxidation. More over, these populations did not have elevated circulatory levels of BCAA to start with. For this reason, I will administrate Na-PB in a population not suffering from urea cycle disorders, but in patients with T2D having elevated systemic levels of BCAA. Therefore, Pheburane will be used off-label in this study. I will use Pheburane as a tool to investigate the potential beneficial effects of enhanced BCAA oxidation and lower circulatory BCAA levels on metabolic health in patients with T2D.

Pheburane is a ‘hybrid medicine’. This means that it is similar to a ‘reference medicine’ containing the same active substance, but Pheburane granules are available at a lower strength and contain different excipients (inactive ingredients) to mask the unpleasant taste of the active substance. In this way compliance towards the use of Na-PB is higher. The reference medicine for Pheburane is Ammonaps.

Pheburane will be administered for 2 weeks. In this randomized clinical trail, results will be compared to a placebo, also administered for 2 weeks. By eye, these placebo granules look

identical to Pheburane, except without the presence of Na-PB as active substance. The placebo will contain only the inactive ingredients of the Pheburane granules. The color of all granules is off-white.

5.2 Use of co-intervention (if applicable)

Participants are advised to maintain their normal sleeping and activity pattern. Their normal dietary habits will be maintained in order to minimize the risk for low levels of glutamine, nitrogen or BCAA. According to the SPC text for Pheburane, the following medicinal products could better be avoided: Probalan (probenecid), Haldol (haloperidol), Depakene (valproate) and medical products containing corticosteroids, due to the risk of hyperammonaemia (also included in exclusion criteria). However, it should be noted that the risk for hyperammonaemia occurs in patients with urea cycle disorders, a group who will not be included in the study. Furthermore, to reduce the risk on hematomas during muscle biopsies, participants will be asked to avoid taking non-steroidal anti-inflammatory drugs (NSAIDs) 3 days preceding taking the muscle biopsies (day 1 and 15).

The use of the participants' regular medication will be checked by the dependent medical doctor and he decides whether this medication would interfere with the study outcome parameters. All medication used by the participants will be reported in the electronic case report form (eCRF).

Two days prior to the visits 2, 3, 5 and 6, participants will be asked to perform no extensive physical activity other than daily routine activities. This includes no performance of any sport and performing walking/ cycling no longer than 30 minutes at low intensity only. Participants should refrain as well from heavy 'household' tasks, like cleaning the windows, gardening or lifting heavy groceries.

5.3 Escape medication (if applicable)

Not applicable.

6. INVESTIGATIONAL PRODUCT

6.1 Name and description of investigational product(s)

Sodium-phenylbutyrate (Na-PB):
Na-PB forms the active substance of Pheburane. Phenylbutyrate is known to be oxidized to phenylacetate, which is enzymatically conjugated with glutamine to form phenylacetylglutamine in the liver and kidneys (**figure 7)**.

Na-PB activates the BCKD complex, the rate limiting enzyme of the BCAA oxidation. With the activation of BCKD complex activity, BCAA oxidation will be enhanced thereby reducing BCAAs in plasma and muscle.

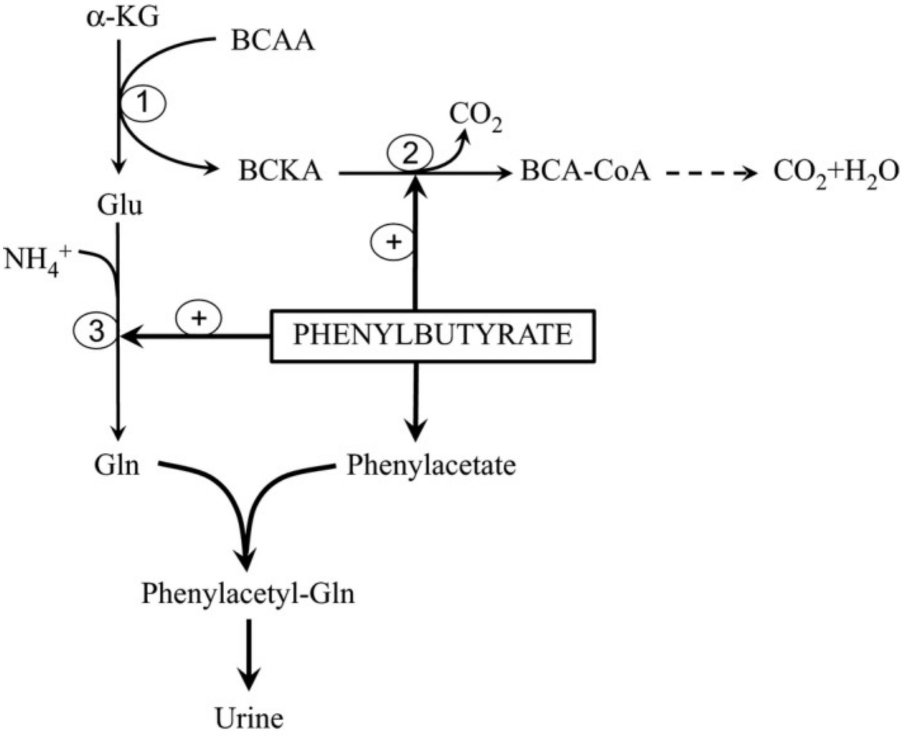

***Figure 7**: scheme demonstrating the role of BCAA in GLN synthesis and the supposed effects of PB administration on GLN and BCAA metabolism. 1, BCAA aminotransferase; 2, BCKA dehydrogenase; 3, glutamine synthetase*

In more detail, Na-PB inhibits the kinase regulating the BCKD complex activity via phosphorylation of the E1alpha subunit (**figure 8**). The phosphorylation of the kinase will alleviate inhibition of the BCKD complex, thereby promoting BCAA oxidation [24, 39].

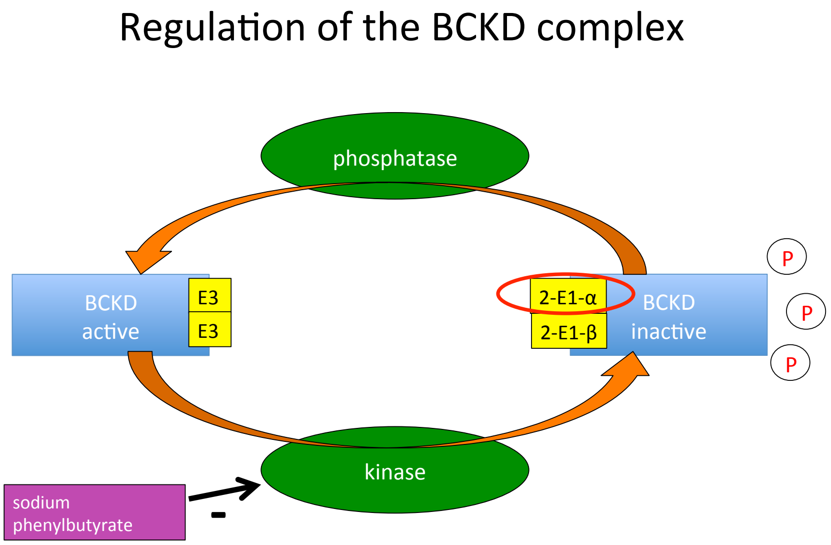

***Figure 8**: enzymes involved in BCAA metabolism including the brached-chain amino acid dehygrogenase (BCDH). Sodium phenylbutyrate inhibits the kinase resulting in less phosphorylation of the 2-E1-alpha subunit of the BCKD complex.*

Phenylacetate is also hydrolyzed by esterases in the liver and blood. Phenylbutyrate is rapidly absorbed under fasting conditions. After a single oral dose of 5 g of Na-PB in the form of Pheburane granules, measurable plasma levels of phenylbutyrate were detected 15 minutes after dosing. The elimination half-life was estimated to be 0.8 hours. Measurable plasma levels of phenylacetate and phenylacetylglutamine were detected 30 and 60 min respectively after dosing. The mean time to peak concentration was 3.55 and 3.23 hours, respectively, and the main peak concentration was 45.3 and 62.8 µg/ml, respectively. The elimination half-life was estimated to be 1.3 and 2.4 hours, respectively. Aproximately 80-100% of the medicinal product is excreted by the kidneys within 24 hours as the conjugated product, phenylacetylglutamine.

1 gram of Pheburane granules contains 483 mg of Na-PB, the active substance. The rest consist of inactive ingredients mainly sucrose and maize starch, but will also contain hypromellose, ethylcellulose N7, macrogel 1500 and povidone K25.

Placebo:
The placebo granules contain only the inactive ingredients of the Pheburane granules, which are mainly sucrose and maize starch, a similar amount of sodium (124 mg), but also hypromellose, ethylcellulose N7, macrogel 1500 and povidone K25. The placebo will be manufactured via Tiofarma, who will provide us with a detailed IMPD.

6.2 Summary of findings from non-clinical studies

Not applicable.

6.3 Summary of findings from clinical studies

Here I would like to refer to the content of the SPC text of Pheburane in which information is provided on pharmacokinetics and preclinical safety data obtained from research published in peer-reviewed articles.

Nitrogen balance and Na-PB treatment

Administration of Na-PB promotes urinary nitrogen disposal, in fact this is the reason why this drug is FDA approved for use in patients having urea cycle disorders. The question is whether or not Pheburane can introduce a negative nitrogen balance in a population without having urea cycle disorders. This has been investigated healthy human subjects who underwent a multitracer study [27]. This study revealed that Na-PB did not result in a negative nitrogen balance. In line, results from a clinical trial reported no adverse events on nitrogen balance, neither glutamine depletion, for patients with thalassemia who were administered with Na-PB between 41 up to 460 days [40]. For patients with T2D studies examining protein degradation and/or synthesis results are not unequivocal [41-43]. If patients with T2D happen to have compromised protein synthesis or increased protein degradation rates, development of a negative nitrogen balance cannot *a priori* be excluded upon administration of Na-PB. Although we do not expect a negative nitrogen balance or glutamine depletion based on all the data reported in healthy people as well as diverse patients groups, to be at the safe side, nitrogen balance will be monitored weekly, along with plasma glutamine levels. Based upon this, nitrogen balance can be restored with supplementation, which will be started only if indicated. According to the SPC text, supplementation of single amino acids to restore nitrogen balance during the use of Na-PB has been recommended for patients having urea cycle disorders. Based on this, the effectiveness of the drug would not be hampered.

BCAA plasma levels and NaPB treatment

Na-PB has been successfully applied as a BCAA lowering agent in MSUD patients [38], as well as in healthy subjects possessing normal BCAA levels before Na-PB was administered [24-28]. More specifically, in healthy people Na-PB induced a drop in leucine ranging from 26-51%, a drop in isoleucine between 33-50% and a drop in valine round 30%. Moreover, it is of relevance to note that the mode of action of Na-PB is via activation of the BCKD complex. In T2D, the activity of this enzyme in skeletal muscle is reportedly compromised by some 63% [20] leaving ample room for improvement.

To support the marketing authorisation for the use of Pheburane, there is one bioequivalence study with cross-over design under fasting conditions. This study was the pivotal study for the marketing authorization application. Pheburane was tested against its reference medicinal product: AMMONAPS. Compared to the FDA-approved Ammonaps, Pheburane contains bad taste-masking inactive ingredients. The two formulations were tested and compared for taste and bioequivalence in healthy volunteers. Volunteers consumed a single dose of the two formulations of sodium phenylbutyrate granules 5 grams (5.32 gram Ammonaps vs. 10.21 gram Pheburane). This study was conducted at the Bloemfontein Early Phase Clinical Unit at the University of the Free State, South Africa. The study consisted in two treatment periods separated by a washout period of a week. Based on the presented bioequivalence study Pheburane is considered bioequivalent with Ammonaps. Therefore, no new pharmacodynamics studies –as already performed for authorization for Ammonaps- are required for Pheburane. For other results, please see 'Assessment Report Pheburane' by the CHMP (see document D2).

6.4 Summary of known and potential risks and benefits

- excess urinary loss of nitrogen and a related negative nitrogen balance: if indicated, nitrogen balance will be restored with supplementation.
- adverse reactions like loss of appetite and changed body odor: this can be caused by phenylacetate and reduced taste perception has been described for 3-4% of all patients with prolonged prescription. These reactions could compromise compliance, therefore, a dropout of ~20% is anticipated.
- amino acid deficiency: participants will be advised to keep their normal dietary habits, to exclude this risk.
- for detailed list of adverse reactions, please see SPC text page 4-5.

In general, to avoid eventual adverse events, patients with congestive heart failure and and/or severe renal insufficiency or known sodium retention with oedema will not be included in the study. Plasma levels of ammonia, arginine, essential amino acids (especially branched chain amino acids), carnitine and serum proteins will be monitored and need to be within normal limits. Plasma glutamine should be maintained at levels less than 1,000 µmol/L. The nitrogen balance will be monitored by use of 24-hour urine samples. Body mass will be measured weekly as well. In our laboratory setting, which is completely dedicated to run these kind of complicated human trials, monitoring of the patients by a physician is routinely embedded. Safety monitoring occurs continuously and any adverse event will be documented and discussed with the responsible physician. Other

eventual adverse events, who are not mentioned in the SPC text, will be reported at lareb.nl. Pheburane (and its reference medicinal product Ammonaps) is safe to use, when not exceeding the dose of 20 g NaPB /day (above this dose no test results are available). As mentioned in the SPC text, a total dose of ~10 g NaPB /day Pheburane may be required and used life-long unless orthotropic liver transplantation is elected in patients with urea cycle disorders. In this study, a lower daily dose of NaPB will be used (see 6.5) for 15 days. Therefore, it is safe for the participants to use Pheburane. No direct benefits in this study are expected for the participants.

6.5 Description and justification of route of administration and dosage

The usual total daily dose of Pheburane in clinical practice is 9.9-13.0 g NaPB /m^2 /day in adults. In this study, 4.8 g NaPB /m^2 /day or 9.94 g Pheburane/m^2/day will be administered to patients with T2D, which is below the lowest dose of clinical prescription for treatment of urea cycle disorders. For every participant daily dose will be calculated according a specific drug calculation file. On average, the overweight/obese participants will have a body surface area (BSA) ~2.1 m^2, meaning a total prescription of ~21g Pheburane a day. The total daily dose needs to be administered orally, spread over the day with each meal (3 times ~7 g per day).

6.6 Dosages, dosage modifications and method of administration

Pheburane will be administered in the form of granules. The granules should be taken orally. The total daily dose should be divided into equal amounts and given with each meal (3 times a day). The granules can be directly swallowed with a drink (water, fruit juices) or sprinkled on to a spoonful of solid foods (mashed potatoes or apple sauce). In the latter case, it is important that it is taken immediately in order to preserve the taste-masking. A calibrated dosing spoon is provided which dispenses up to 3.7 g by graduation of 740 mg.

6.7 Preparation and labelling of Investigational Medicinal Product

The study drug will be packaged by Tiopharma. Labels will be prepared in accordance with GMP and local regulatory guidelines. The labels will fulfil GMP Annex 13 requirements for labelling. This means that the label should contain:

- information to identify contact person or persons involved in the clinical trial (researcher: Froukje Vanweert)
- information to identify the clinical trial (NTR7426; EudraCT: 2018-003176-13)
- randomisation code to identify the medicinal product if needed
- information related to the use of the medicinal product (see document D3.)

The label text will be in Dutch. The study drug will be kept in a secure place with appropriate storage conditions at the research facility, kept at room temperature.

Importantly, the study will be performed in a double-blinded manner. Therefore, the study drug and placebo will be supplied in identical bottles thereby enabling double-blind conditions.

6.8 Drug accountability

All study medication will be packed, blinded and labelled by Tiopharma. Tiopharma will send the study medication to the hospital pharmacy (MUMC+) who will send the medication to the researcher (Froukje Vanweert). The researcher will weigh the bottles filled with study medication (granules) before given it to the participants. The weight of the bottles will be noted in a log. Participants are asked to return the used bottles at the end of each treatment period. The weight of the returned bottles will be noted as well in the log. Participants should return all unused study drugs and/or empty bottles to the researcher. The study drug of participants who finalized the study will be destroyed with the hospital pharmacy. Each dispensation of the study drug will be documented in the eCRF.

7. NON-INVESTIGATIONAL PRODUCT

Stable isotope [6,6-$^{2}H_2$] glucose

The stable isotope [6,6-$^{2}H_2$] glucose (approved IMPD within several previous studies performed by our research group and approved by the METC: protocol ID 16-3-019, 15-3-046, 15-3-030, 13-3-040, 09-3-039, 09-3-033) will be infused during the clamp to make it possible to measure rates of glucose appearance and disappearance (IMPD of [6,6-$^{2}H_2$] glucose attached in document D2)

7.1 Name and description of non-investigational product(s)

- Lidocainehydrochloride 1%
- Novorapid 100 IE/ml (Novo Nordisk)
- Glucose 20% (Baxter)
- Stable isotope [6.6-$^{2}H_2$] glucose

7.2 Summary of findings from non-clinical studies

Not applicable.

7.3 Summary of findings from clinical studies

- Please find SPC for Lidocainehydrochloride 1% (document D2)
- Please find SPC for Novorapid 100 IE/ml (document D2)
- Please find SPC for Glucose 20% (document D2)
- Please find IMPD for Stable isotope [6.6-$^{2}H_2$] glucose (document D2)

7.4 Summary of known and potential risks and benefits

- Please find SPC for Lidocainehydrochloride 1% (document D2)
- Please find SPC for Novorapid 100 IE/ml (document D2)
- Please find SPC for Glucose 20% (document D2)
- Please find IMPD for Stable isotope [6.6-$^{2}H_2$] glucose (document D2)

7.5 Description and justification of route of administration and dosage

All applied as in standard medical practice.

7.6 Dosages, dosage modifications and method of administration

A 1 IE/ml insulin infusion for intravenous administration during the clamp will be prepared using 0.5 ml of 100 IE/ml Novorapid insulin, diluted in 47.5 ml NaCl 9% and 2 ml blood from the participant. Preparation will be checked and co-singed by a second experienced researcher.

A total of 64.5 ml of [6.6-$^2H_2$] glucose isotopic tracer and 5 ml KCl 7.5% is added to a 500 ml of glucose 20% infusion bag, to prepare the glucose solution for intravenous administration during the clamp. The solution will be administered at variable infusion rates in order to keep glucose values euglycemic, which means around 5 mmol/l. This prepared 20% glucose infusion bag will be carefully marked by the researcher and checked/co-singed by a second experienced researcher.

A primed (2.4 mg/kg) continuous (0.04 mg/kg/min) intravenous infusion of [6.6-$^2H_2$] glucose isotopic tracer will be administered throughout the duration of the clamp. The ampules contain ‘ready to use’ [6.6-$^2H_2$] glucose isotopic tracer, which will be transferred into a 50 ml syringe, carefully marked by the researcher and checked/co-singed by a second experienced researcher.

Prior to the muscle biopsy, between 5 to 10 ml of Lidocainehydrochloride 1% is applied as anaesthesia to the subcutaneous and (sub) fascial tissue.

7.7 Drug accountability

Lidocaine, insulin, glucose 20% and isotopic tracers are used and ordered at regular base for many studies. Lidocaine, insulin and glucose 20% are ordered with the MUMC pharmacy and the glucose isotopic tracer are ordered via the Radboud pharmacy in Nijmegen. All products arrive in their original packaging and are stored appropriately.

8. METHODS

8.1 Study parameters/endpoints

8.1.1 Main study parameter/endpoint

Whole-body insulin sensitivity: insulin sensitivity will be expressed during the clamp as insulin-stimulated rate of glucose disappearance (ΔRd) given in µmol/kg/min. Insulin sensitivity index during an OGTT expressed as glucose clearance in ml/kg/min.

8.1.2 Secondary study parameters/endpoints (if applicable)

- muscle mitochondrial function expressed as O_2-flux in pmol/mg/s
- whole-body energy metabolism expressed as respiratory exchange ratio (RER) and kJ/kg/min
- fat accumulation in muscle and the liver expressed as %

8.1.3 Other study parameters (if applicable)

Baseline participant characteristics, such as age, gender, physical performance, smoking, alcohol consumption, BMI, body mass, fasting HbA1c, fasting plasma glucose, fasting plasma insulin, fasting plasma free fatty acids, muscle mass and body composition (% fat mass and % fat free mass). Furthermore, amino acids profile in plasma.

8.2 Randomisation, blinding and treatment allocation

When participants come to the research facility for the screening, an anonymous code will be randomly assigned. Anonymous codes will be obtained via the website www.randomizer.org. After participants have successfully past the screening, they will be randomly allocated to one of the two interventions groups (A or B) using ‘controlled randomization’. This type of randomization will be used in order to ensure that the order in which the interventions are given is the same for both interventions to prevent possible confounding order effects. Tiopharma performs another randomization in which A or B are allocated to placebo or Pheburane. Per participant A can be placebo or Pheburane. In this way in case of necessary unblinding, afterwards, the study team will still be blinded. The researcher via www.randomizer.org will do the randomization to allocate participants to group A or B. First, randomization will be done for 9 participants with 2 treatment arms (Pheburane and placebo). When a total of 9 participants have completed the study, interim analysis will be performed (see section 10.4 interim analysis). The study will be continued when the results of the interim analysis are known and approved by the METC. Thereafter, 9 other participants will be randomized within 2 treatment arms. This randomisation list containing the anonymous participant codes and allocation to the treatment A or B will be

send to Tiopharma. Accordingly, Tiopharma prepares the study bottles for the participants. For every participant Tiopharma prepares four envelops which contain the unblinding key for the intervention arms A and B. One envelope will be sent to the principle investigator (Dr. Esther Phielix), one to the MUMC+ hospital pharmacy (Rogier van der Zanden), one to the secretary of the Nutrition and Movement Sciences department (Yolanda Verhagen) and one to the responsible medical physician of the study (Dr. T. van de Weijer). All these players are responsible to keep these envelopes safe. The envelopes will be opened only in situations where de-blinding of the study is necessary. In view of the nature of this study no indications for breaking the randomization code are predetermined, neither expected. In a remote possibility of any untoward effect, the persons mentioned above can break the blinding code for the particular participant.

In case of emergency, using this way of blinding strategy, the study team remains blinded upon opening the envelope as A will not be always placebo or Pheburane. Tiopharma will provide the study drug in labelled bottles containing the necessary information of the study (see paragraph 6.7).

The randomisation file containing the anonymous participant codes and the randomization list with the allocation to group A or B will be saved with a password. This file can be opened at any time by the research team (researcher: Froukje Vanweert; Principle investigator: Dr. Esther Phielix).

8.3 Study procedures

A detailed time line for this study is depicted in **figure 9**.

Screening

If interested in participating the study, participants will receive detailed information by email or postal services accompanied by a general brochure provided by the Dutch government about participating in a medical study. The participant will be instructed to read it carefully and to ask questions if anything is unclear. We ask the participant if the researcher may contact the participant again after 7 days of consideration to answer any remaining questions. Only when the informed consent is signed, participants can undergo the screening. Both the participant as well as the researcher will sign the informed consent at side before the start of the screening. The screening will include the following handlings:

- together with the participant, a questionnaire will be filled on health status, physical activity and medical history (see document F1.1)

- a fasting blood sample will be drawn to determine clinical health parameters including parameters for liver and kidney function
- physical examination, like body weight and length
- blood pressure will be measured 3 times in row at the contra-dominant arm

A total of 17 ml blood will be drawn.

Visit 1 *and* ***visit 4*** *(day 1 AND day 57; duration 1 hour)*

Participants will arrive upon an overnight fast in the morning. A **blood sample** will be drawn to determine baseline concentrations of ammonia, amino acids (including arginine and essential amino acids), total protein, potassium, glucose, free fatty acids, and triglycerides. A total of 18 ml blood will be drawn. At the end of the visit the study medication will be provided and the participant will be orally informed in detail on the route, doses and frequency of administration. The participant will receive this information as well in written in a patient card (see document F3.). The participant will receive breakfast while they take their study medication.

Visit 2 *and* ***visit 5*** *(day 3 AND day 59; duration 3.5 hour)*

Participants will arrive at the research facility in a fasting condition in the morning. First, upon placement of an intravenous cannula, a **blood sample** will be drawn to determine baseline concentrations of ammonia, amino acids (including arginine and essential amino acids), total protein, potassium, glucose, free fatty acids and triglycerides. A total of 9 ml blood will be drawn. Second, a **muscle biopsy** will be taken from the m. vastus lateralis performed under local anaesthesia with Lidocaine 1% solution (10 mg/ml) (without adrenaline). A side-cutting needle will be used to acquire the muscle tissue, following the Bergström method (SOP provided in document K6) [44]. The leg of the biopsy will be randomized to exclude the influence of relatively more trained muscle versus a relatively less trained muscle. The biopsy material will be processed *ex vivo* immediately. One portion will be assayed using the high-resolution respirometry to measure mitochondrial function. High-resolution respirometry will be performed using a two chamber oxygraphy (OROBOROS Instruments). Multiple substrate/inhibitor titration protocols will be applied to extensively characterize the mitochondrial capacity of skeletal muscle. The remaining material will be frozen in isopentane cooled with liquid nitrogen and stored at minus 80 °C for later analyses. For these analyses, about 300 mg of muscle tissue is needed, being a standard amount of tissue that is to be acquired in each biopsy. Third, indirect calorimetry will be performed using the ventilated hood for 30 minutes. Fourth, a two-hour oral glucose tolerance test (OGTT) will be used to calculate insulin sensitivity. Two days before the OGTT, participants will

be asked to refrain from strenuous exercise. A standardized 2h OGTT will be performed. A catheter will be placed in the antecubital vein and fasting blood samples will be collected, and 30, 60, 90 and 120 minutes after the ingestion of the 75 gram of glucose solution, a total of 25 ml blood will be collected. After the OGTT, the participants will receive breakfast while they take their study medication.

***Visit 3** and **visit 6** (day 14 till day 15 AND day 71 till day 72; duration 24 hours)*

Day 14 and Day 71

At this day participants are advised to have an early lunch at home round 12.00h. Thereafter participants remain fasting. Participants come to the research unit round 16.30 pm. First, they will start to collect their urine for 24 hours to calculate nitrogen balance. The subjects will be provided with a urine container, which need to be handed in after finishing the hyperinsulinemic-euglycemic clamp (day 15 and day 72). The 24-hour urine will be collected in containers by the subject in the preceding 24-hours of this visit. After handing in the urine container, the total volume and molecular weight of urine will be measured and nitrogen balance will be calculated.

Thereafter, participants undergo a measurement in which body composition will be determined. The **BodPod**® (Cosmed) will be used to determine body composition and measures fat mass, fat free mass, total body mass and estimates resting metabolic rate (RMR) via whole-body densitometry. The Bod Pod utilizes of displacement during the measurement to determine body composition. The Bod Pod technology is fundamentally the same as the underwater (hydrostatic) weighing, but uses air instead of water. The volume of air a person’s body displaces is measured while sitting inside a comfortable chamber for two 50- second measurements. It is a safe, non-invasive, easy-to-use and quick tool for measuring body composition. The duration of the measurement is approximately 5 minutes.

After the BodPod measurement, around 17.00 pm, participants will undergo **proton magnetic resonance spectroscopy (^{1}H-MRS)**. This will be used to determine intramuscular and intrahepatic lipid content and upper leg skeletal muscle volume. The volunteers will be positioned with the leg in the MRI scanner and MRI images will be acquired. Thereafter, a volume of interest will be selected within the m. tibialis anterior from which ^{1}H-MRS spectra will be acquired. The water signal dominates the spectra, which will be suppressed using frequency-selective pre-pulses and the spectra will be fitted to quantify the lipid peak. The acquisition will be repeated without water suppression in order to quantify the water peak, which will be used as reference. The

lipid vs. water ratio will be used as parameter of lipid content. For measurements in the liver, the volunteers will be repositioned and MRI images of the abdomen will be acquired. Thereafter, a volume of interest will be selected within the right lobe. The ^{1}H-MRS spectra will be acquired from this selected region. To prevent motion artefacts, patients will be asked to breathe in the 4s-rhythm of the spectroscopic measurement for about 6 minutes. Also here, the water signal is dominating the proton spectra and will be suppressed using frequency-selective pre-pulses to quantify the lipid peak. The lipid/water ratio will be used as the parameter of intrahepatic lipid content. Total acquisition time (with repositioning of patient and imaging) should be about 90 minutes.

Upon finishing the MRS measurement, participants consume a standardized meal at the research facility before going into the **respiration chamber** for 12 hours. During the overnight stay, the sleeping metabolic rate (SMR), diet-induced thermogenesis, and substrate oxidation will be measured in the respiration chamber using indirect calorimetry equipment (Omnical, Maastricht, the Netherlands), which measures concentrations of oxygen consumption and carbon dioxide production. The respiration chamber is a 14 m^2 room furnished with a bed, chair, desk, TV, telephone, computer, washbowl and toilet. The room is ventilated with fresh air Privacy is warranted, as the subject in the chamber can close the curtains of the outside windows.

Day 15 and 72

A two-step **hyperinsulinemic euglycemic clamp** will be performed at the end of each study period for the determination of insulin sensitivity according to the method described by DeFronzo et al. [45]. The clamp will be performed in the post-absorptive state at 6.30 am after an overnight fast. A teflon cannula will be inserted into antecubital veins of one arm for the infusion of glucose tracer, insulin and glucose. Another cannula will be inserted retrogradely into a superficial dorsal hand vein. This venous blood will be arterialized by placing the hand into a hotbox, which blows warm air (50°C). A fasting blood sample will be collected. Out of the fasted blood sample, DNA will be extracted from leucocytes in this sample to study genetic variants that may affect hormones and energy metabolism. The results will not have any consequence for the future health condition of the participants, does not have any consequences for the family and is not used for diagnosis or medical treatment. Therefore, the patient will not be informed about the DNA results.

After taking fasting blood samples, a primed continuous infusion of [6,6 $^{2}H_2$] glucose (priming dose: 2.4 mg/kg; continuous infusion: 0.04 mg/kg/min) will be administered (t

=0 min). This is a naturally occurring isotope, which is in no way harmful to humans and will be obtained from the pharmacy of the Academic Hospital Nijmegen.

At t=120 a muscle biopsy will be taken from the *m. vastus lateralis.* After 150 minutes of isotopic equilibration, four blood samples will be obtained at 10-min intervals (t=150, 160, 170 and 180 min) for the determination of basal blood substrates, whole body glucose disposal and hepatic glucose production. Indirect calorimetry (ventilated hood) will be performed during this last half hour of the baseline equilibration period to determine substrate oxidation (t =150 - 180).

At t= 180 minutes, a 3-h low primed constant infusion of insulin is started (10 mU/m^2/min, Novorapid, Novo Nordisk) to asses hepatic insulin resistance. Plasma glucose levels will be clamped at ~5 mmol/L by variable co-infusion of 20% glucose. At regular time points (every 5 to 10 minutes), a small volume of blood (0.9 ml) will be sampled for immediate determination of plasma glucose concentration. When necessary, glucose infusion rate will be adjusted to obtain plasma glucose levels of ~5.0 mmol/L (euglycemia). Indirect calorimetry will be performed at t =330-360 min. During this time period, four blood samples will be obtained at 10-min intervals for the determination of whole-body glucose disposal and hepatic glucose production (t =330, 340, 350 and 360). At t=360 the 2,5-h high primed constant insulin infusion is started (40 mU/m^2/min), in order to fully stop the hepatic glucose production and only study the rate of disappearance (Rd) as a measure for skeletal muscle insulin sensitivity. Here again plasma glucose levels will be clamped at ~5 mmol/L by variable co-infusion of 20% glucose. At regular time points (every 5 to 10 minutes), a small volume of blood (0.9 ml) will be sampled for immediate determination of plasma glucose concentration. When necessary, glucose infusion rate will be adjusted to obtain plasma glucose levels of ~5 mmol/L (euglycemia). Thirty minutes after the start of the high insulin infusion (at t = 390), a second muscle biopsy will be taken. Indirect calorimetry will be performed at t=510-540 min. During this time period, four blood samples will again be obtained at 10-min intervals (t =480, 490, 500 and 510). During the indirect calorimetry during the clamp, respiratory gas exchange will be measured using an open-air circuit respirometry with an automated ventilated hood system (Omnical, Maastricht Instruments, Maastricht University, Maastricht, The Netherlands). At the end the participants go home after handing in their 24-hour urine collection.

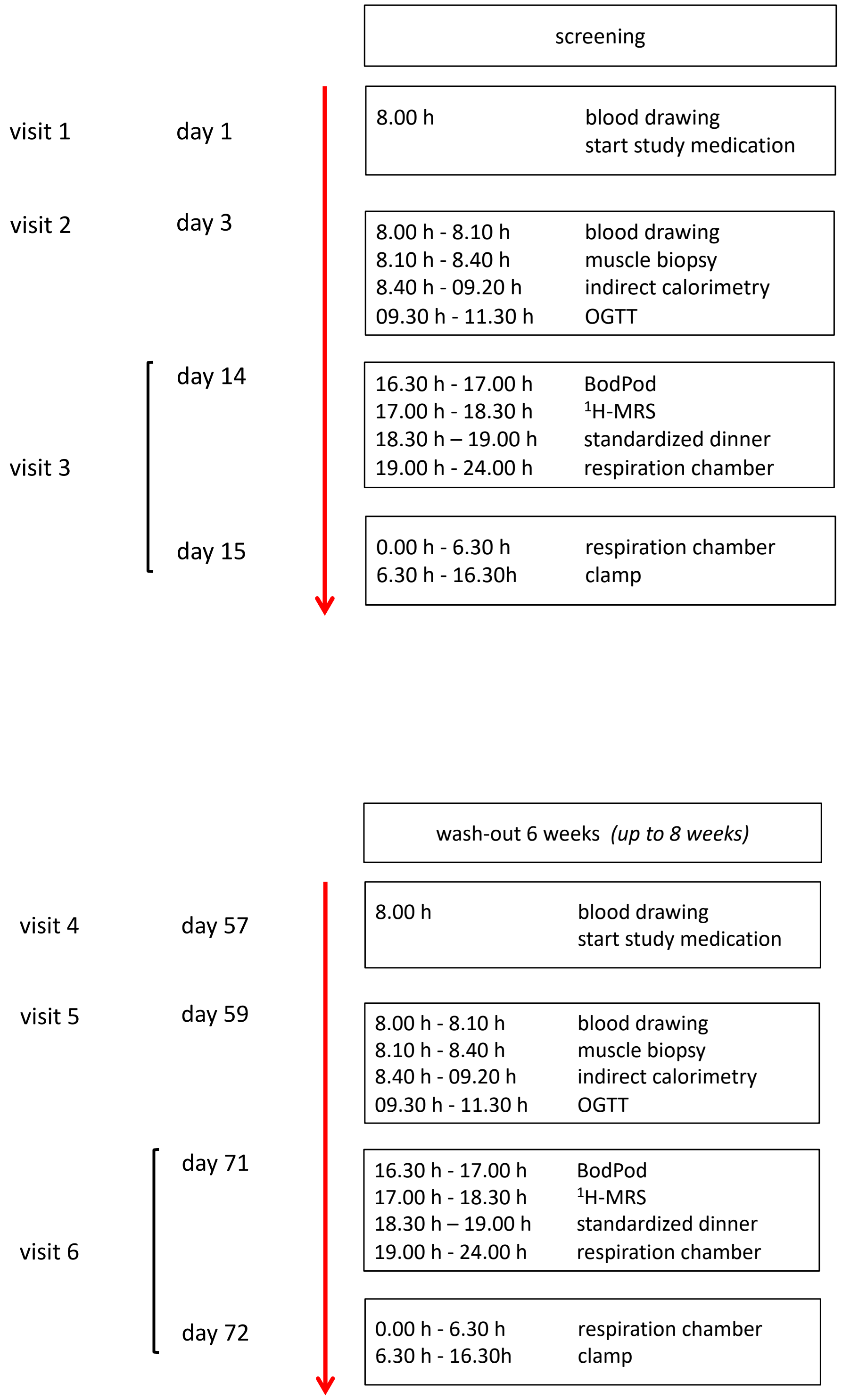

END OF STUDY

***Figure 9**: detailed time table of the RCT including the screening, two treatments period, washout and end of study. During visit 3 and 6, a 24-hour urine will be collected by the subject in the preceding 24-hours of this visit.*

8.4 Withdrawal of individual subjects

Subjects can leave the study at any time for any reason if they wish to do so without any consequences. The investigator can decide to withdraw a subject from the study for urgent medical reasons.

8.4.1 Specific criteria for withdrawal (if applicable)

The investigator can decide to withdraw a participant from the study for the following reasons:

- non-medical reasons e.g. request by the participant or non-compliance to the study
- medical reasons argued to be significant by the medical responsible doctor/research and/or participant
- protocol violation
- in case of illness or changed use of medication of the participant

In all cases, the researcher together with the medical responsible doctor decides whether the participant may continue the study or not.

8.5 Replacement of individual subjects after withdrawal

Participants who withdraw from the study will not be replaced. Withdrawal has been anticipated for within the sample size calculation.

8.6 Follow-up of subjects withdrawn from treatment

Upon withdrawal, no follow-up of the participant will take place. If withdrawal occurs due to medical complications, the participant will be guided to the responsible medical doctor of the study and eventually be referred to the general practitioner

.

8.7 Premature termination of the study

The study may be premature terminated if:

a) the judgment of the competent medical research ethics committee that has assessed the study is irrevocably revoked;

b) a reasonable case can be made for terminating the study in the interests of the health of the research subjects;

c) it transpires that continuation of the study cannot serve any scientific purpose, and this is confirmed by the medical research ethics committee that has issued a positive decision on the study;

d) the principal investigator is no longer capable of performing the tasks of the principal investigator, and no replacement can be found;

e) circumstances beyond the control of the investigator make it impossible or unnecessary to continue the study

9. SAFETY REPORTING

9.1 Temporary halt for reasons of subject safety

In accordance to section 10, subsection 4, of the WMO, the sponsor will suspend the study if there is sufficient ground that continuation of the study will jeopardise subject health or safety. The sponsor will notify the accredited METC without undue delay of a temporary halt including the reason for such an action. The study will be suspended pending a further positive decision by the accredited METC. The investigator will take care that all subjects are kept informed.

9.2 AEs, SAEs and SUSARs

9.2.1 Adverse events (AEs)

Adverse events are defined as any undesirable experience occurring to a subject during the study, whether or not considered related to the investigational product, trial procedure and/or the experimental intervention. All adverse events reported spontaneously by the subject or observed by the investigator or his staff will be recorded. Other eventual adverse events, who are not mentioned in the SPC text, will be reported at lareb.nl.

9.2.2 Serious adverse events (SAEs)

A serious adverse event is any untoward medical occurrence or effect that

- results in death;
- is life threatening (at the time of the event);
- requires hospitalisation or prolongation of existing inpatients' hospitalisation;
- results in persistent or significant disability or incapacity;
- is a congenital anomaly or birth defect; or
- any other important medical event that did not result in any of the outcomes listed above due to medical or surgical intervention but could have been based upon appropriate judgement by the investigator.

An elective hospital admission will not be considered as a serious adverse event.

The investigator will report all SAEs to the sponsor without undue delay after obtaining knowledge of the events.

The sponsor will report the SAEs through the web portal *ToetsingOnline* to the accredited METC that approved the protocol, within 7 days of first knowledge for SAEs

that result in death or are life threatening followed by a period of maximum of 8 days to complete the initial preliminary report. All other SAEs will be reported within a period of maximum 15 days after the sponsor has first knowledge of the serious adverse events.

9.2.3 Suspected unexpected serious adverse reactions (SUSARs)

Adverse reactions are all untoward and unintended responses to an investigational product related to any dose administered.

Unexpected adverse reactions are SUSARs if the following three conditions are met:

1. the event must be serious (see chapter 9.2.2);
2. there must be a certain degree of probability that the event is a harmful and an undesirable reaction to the medicinal product under investigation, regardless of the administered dose;
3. the adverse reaction must be unexpected, that is to say, the nature and severity of the adverse reaction are not in agreement with the product information as recorded in:
    - Summary of Product Characteristics (SPC) for an authorised medicinal product;
    - Investigator’s Brochure for an unauthorised medicinal product.

The sponsor will report expedited the following SUSARs through the web portal *ToetsingOnline* to the METC:

- SUSARs that have arisen in the clinical trial that was assessed by the METC;
- SUSARs that have arisen in other clinical trials of the same sponsor and with the same medicinal product, and that could have consequences for the safety of the subjects involved in the clinical trial that was assessed by the METC.

The remaining SUSARs are recorded in an overview list (line-listing) that will be submitted once every half year to the METC. This line-listing provides an overview of all SUSARs from the study medicine, accompanied by a brief report highlighting the main points of concern.

The expedited reporting of SUSARs through the web portal Eudravigilance or ToetsingOnline is sufficient as notification to the competent authority.

The sponsor will report expedited all SUSARs to the competent authorities in other Member States, according to the requirements of the Member States.

The expedited reporting will occur not later than 15 days after the sponsor has first knowledge of the adverse reactions. For fatal or life threatening cases the term will be maximal 7 days for a preliminary report with another 8 days for completion of the report.

9.3 Annual safety report

In addition to the expedited reporting of SUSARs, the sponsor will submit, once a year throughout the clinical trial, a safety report to the accredited METC, competent authority, and competent authorities of the concerned Member States.
This safety report consists of:

- a list of all suspected (unexpected or expected) serious adverse reactions, along with an aggregated summary table of all reported serious adverse reactions, ordered by organ system, per study;
- a report concerning the safety of the subjects, consisting of a complete safety analysis and an evaluation of the balance between the efficacy and the harmfulness of the medicine under investigation.

9.4 Follow-up of adverse events

All AEs will be followed until they have abated, or until a stable situation has been reached. Depending on the event, follow up may require additional tests or medical procedures as indicated, and/or referral to the general physician or a medical specialist. SAEs need to be reported till end of study within the Netherlands, as defined in the protocol

9.5 Data Safety Monitoring Board (DSMB) / Safety Committee

Pheburane is a FDA-approved drug with well-described side effects or risk (see SPC text Pheburane and paragraph 6.4 and 13.1.e). In several studies, Na-PB has been administered in healthy humans and in people diagnosed with a disease (MSUD, urea cycle disorders, thalassemia) [24-28, 40]. No additional risks are expected with the administration of Pheburane in patients with T2D. In this study, we will use Pheburane in a dose which is below the lowest dose of clinical prescription for only 2 weeks. Pheburane will be used as a tool to boost BCAA oxidation in patients with T2D (off-label).

10. STATISTICAL ANALYSIS

Visual inspection of data histograms and Kolmogorov-Smirnov normality tests will be performed to evaluate normal distribution. In case the data is normally distributed a parametric test will be applied to test for statistical differences between Pheburane and placebo conditions. In case the data is not normally distributed a non-parametrical test will be applied or the data will be transformed as appropriate (for example Log transformation).

Descriptive data

Data will be presented as minimum, maximum, mean and standard deviation or standard error. The hypothesis being tested is:

$H_0 : \mu_{placebo} = \mu_{pheburane}$

$H_A : \mu_{placebo} \neq \mu_{pheburane}$

Statistics will be performed using SPSS 21.0 for MAC and a two-sided $p<0.05$ will be considered statistically significant.

10.1 Primary study parameter(s)

The primary study parameter is insulin sensitivity measured as glucose infusion rate (GIR) expressed as µmol/kg/min. During the clamp, four sequential blood samples within a time period of 30 minutes will be taken throughout three test phases (basal phase –which is before the start of the insulin-, low-insulin phase and high-insulin phase). The average of these four blood samples per phase will be averaged to further calculate glucose tracer kinetics: rate of appearance (Ra), and rate of disappearance (Rd). Calculations for on-steady state Ra and Rd during the clamp will be performed as described by Wolf et al. [46] and Steele et al. [47]. Endogenous glucose production (EGP) will be calculated as Ra minus GIR. The Rd, Ra, EGP and GIR from each phase of the test will be compared between Pheburane and placebo condition using the parametric paired Student's t-test or the non-parametric Wilcoxon signed rank test. Whether these parameters differ among the different phases of the clamp test (in time) will not be tested. Every state will be tested separately according to international standards.

10.2 Secondary study parameter(s)

All secondary study parameters are numerical variables (e.g. glucose clearance in m/kg/min, liver fat percentage, O2-flux in pmol/mg/s and whole-body energy metabolism in kJ/kg/min). In addition, for some parameters the numerical numbers will be averaged over

a certain time period, e.g. to determine sleeping metabolic rate measured in the respiration chamber in kJ/kg/min, average O_2 consumption and CO_2 production during the ventilated hood measurements over a time period of 30 min. For all parameters, the placebo vs. Pheburane condition will be tested using the parametric paired Student's t-test or the non-parametric Wilcoxon signed rank test.

Linear relationships (associations) between secondary parameters and the primary parameters will be analyzed by calculating the Pearson's correlation coefficient in case data is normally distributed. The non-parametric equivalent to Pearson's correlation coefficient, Spearman's correlation coefficient, will be used in case is not normally distributed or when non-linear relationship is assumed.

10.3 Other study parameters

Participant characteristics like age, BMI, height ect. will not be statistically analysed, however only presented as minimum, maximum, mean and standard deviation. Results of the safety checks (e.g. plasma amino acid values) will be analysed using repeated measures two-way ANOVA and when significant changes occur within time or between Phebureane vs. placebo, a post hoc test will be applied using the paired Student's t-test with Bonferroni correction to specify these differences.

Missing values will not be replaced. If a missing value occurs for a specific parameter, the participant in question will not contribute to the analysis of that parameter.

10.4 Interim analysis

Since this is the first study assessing the effect of Pheburane administration on BCAA metabolism and insulin sensitivity in patients with T2DM, it is difficult to determine the required number of participants needed. To be on the safe side, we estimated that we need to include 18 participants in our study. By performing an interim analysis when half of the group completed the study (n=9), we have more insight about the number of subjecs needed to reach the intended effect size
Early data analysing might prevent unnecessary burden of human participants. Interim analysis will be performed by the PI, using the randomisation codes of the intervention, in order to ensure independent data analysis.

11. ETHICAL CONSIDERATIONS

11.1 Regulation statement

The Medical Ethical Committee of the Maastricht University approved this study. The study will be conducted according to the Declaration of Helsinki (64th WMA General Assembly, Fortaleza, Brazil, October 2013) [48], the Principles of GCP and is in accordance with the Medical Research Involving Human Subjects Act (WMO).

11.2 Recruitment and consent

Subjects will be recruited in Maastricht and surroundings by means of posters, flyers and advertisements in local newspapers and on the internet (see document E3.1 and E3.2). Participants from previous studies will be contacted as well in case they provided written consent for this. To increase recruitment efficiency, potential participants can register at a mailing list. With this list, the researcher can approach individuals who showed interest in participating scientific research.

The researchers will contact individuals who are interested in participating in the study by telephone, only after the individual has sought contact with the researcher on his or her accord first. By contacting responders by telephone first, the burden of travel and time effort is reduced for the potential subject and researchers. In the telephone interview, the goal of the study will be explained, and the basic inclusion criteria will be discussed. When responders are interested, they will receive detailed subject information via e-mail or mail accompanied by a general brochure (provided by the Dutch government) about participating in a medical study. They will be instructed to read this information carefully and to ask questions if things are unclear. The researcher will contact the possible participant again at least 7 days after the study information was received by the participant. If responders want to participate after reading the study information and seem to be eligible, they are invited for a screening. Before the screening measurements take place, the subject needs to sign an informed consent form. Afterwards, the researcher signs the informed consent as well. It is very important that the subjects sign the form first, to limit any potential pressure of the researcher

11.3 Objection by minors or incapacitated subjects (if applicable)

Not applicable

11.4 Benefits and risks assessment, group relatedness

This study carries no benefits for the subjects and carries minor risks for the subjects. The

major burdens consist of a moderate time commitment and multiple biopsies (six muscle biopsies in total). During visit 3 and 6, we will take frequent blood samples, which can form a risk in case of anaemia. However, people with a low Hb will not be included in the study. The total amount of blood that will be taken during one treatment period is ~250 ml. Blood sampling, infusions and the muscle biopsies can cause bruises. Infections or continued bleeding on the other hand are very rare. Hyperinsulinemic euglycemic clamping is a procedure we perform routinely in our laboratory without notable complications. Glucose levels will be monitored during the clamp every 5 – 10 minutes. In rare occasions subjects exhibit symptoms of hypoglycaemia (even if their blood glucose levels are still above 3 mmol/l), which can be counteracted by increasing glucose infusion rate. After successfully performing the clamp blood glucose values will be monitored for an additional 60 minutes with glucose infusion stand-by if glucose levels happen to drop. Solid food and sugar-drinks will be provided directly after finalizing the clamp to avoid the experience of hypoglycaemia. A medical doctor will be stand-by during the whole clamp in case problems related to the clamp will occur.

Due to local anaesthesia, the muscle biopsies are rather painless, however, participants can experience an uncomfortable pressure within the muscle while taking the biopsy. Participants will be informed on this. To minimize the risk for a hematoma, the location of the biopsies will be compressed for approximately 5 minutes after biopsy and a pressure bandage will be used after the muscle biopsy. The place of incision will leave a small scar (~ 5mm). To promote wound healing, the incision will be sealed with sterile steri-strips and a waterproof band-aid. In some cases, a small skin nerve can be damaged, which will cause a temporary dull feeling at the location of incision (maximal 6-12 months).

MRS is a safe procedure, with no known health risk as long as none of the exclusion criteria are met. All MRI images will be screened by a radiologist, therefore there is a chance that an accidental medical finding can be detected, which may require follow-up diagnostics. If the radiologists advise such, the volunteer will be informed and the general practitioner will also be informed.

The possible risks accompanied with the study drug are outlined in chapter 6.4.

This study will lead to novel insight in the effect of enhanced BCAA metabolism on metabolic health and insulin sensitivity in patients with type 2 diabetes.

11.5 Compensation for injury

The sponsor (also) has an insurance which is in accordance with the legal requirements in the Netherlands (Article 7 WMO). This insurance provides cover for damage to research subjects through injury or death caused by the study.
The insurance applies to the damage that becomes apparent during the study or within 4 years after the end of the study.

11.6 Incentives

The subjects will receive a compensation for their contribution to the study. After complete participation, compensation will be 800 euro. Premature termination of the study protocol will result in a reduced compensation, relative to the duration of the participation.
The compensation is based upon a total of 14 euro / hour. This means that a total of 14 euro x 57 hours = 800 euro will be paid for compensation.
Subjects do not receive payment after completing only the screening. Travelling costs will be compensated with a maximum of 0.19 euro/km when travelling by car and complete cover of expenses when travelling by public transport.

12. ADMINISTRATIVE ASPECTS, MONITORING AND PUBLICATION

12.1 Handling and storage of data and documents

At the start of the study, subjects will be assigned an study code (www.randomizer.org) that will not change during the study. This code is linked with the name, address, date of birth, and telephone number of the participant in a password protected file. For all purposes, this code will be used for participant identification. Only members of the project team (Dr. E. Phielix and Drs. F. Vanweert) can access this file, except for the technicians. The privacy of the participants who take part in the study will be protected. This means that the study code will not contain the participants' initials or birth date. The scope of this project is to evaluate metabolic health upon the administration of study drug Pheburane, with focus at insulin sensitivity, energy metabolism, mitochondrial function and liver/muscle fat content. In case data, documents or samples will be analyzed outside the scope of this proposed study, the participants will be asked for permission. When data or samples collected in this study can be used for future research in the scope of this study, no permission will be asked. Participants will have the opportunity to obtain information regarding their own study results. Handling of these data will be done in accordance with the privacy statement (EU General Data Protection Regulation and the Dutch Act on Implementation of the General Data Protection Regulation). Blood samples and muscle biopsies will be analyzed and when possible stored for future analysis. Muscle biopsy and blood samples will be stored in a freezer from the Department of Nutrition and Movement Sciences. All human material, research data and documents will be stored for 15 years. This is mentioned in the informed consent. If participants do not agree to this, they can't participate in this study. After a period of 15 years all the study the material will be destroyed. Only the research team, IGZ, METC and monitors have access to the research data and documents.

12.2 Monitoring and Quality Assurance

Monitoring will be performed via the CTCM. Parallel to this approval procedure with the METC, a monitor will be assigned to the study, a risk analysis will be made and a monitoring plan will be prepared.

12.3 Amendments

Amendments are changes made to the research after a favourable opinion by the accredited METC has been given. All amendments will be notified to the METC that gave a favourable opinion.

A ‘substantial amendment’ is defined as an amendment to the terms of the METC application, or to the protocol or any other supporting documentation, that is likely to affect to a significant degree:

- the safety or physical or mental integrity of the subjects of the trial;
- the scientific value of the trial;
- the conduct or management of the trial; or
- the quality or safety of any intervention used in the trial.

All substantial amendments will be notified to the METC and to the competent authority.

12.4 Annual progress report

The sponsor/investigator will submit a summary of the progress of the trial to the accredited METC once a year. Information will be provided on the date of inclusion of the first subject, numbers of subjects included and numbers of subjects that have completed the trial, serious adverse events/ serious adverse reactions, other problems, and amendments.

12.5 Temporary halt and (prematurely) end of study report

The sponsor will notify the accredited METC and the competent authority of the end of the study within a period of 90 days. The end of the study is defined as the last patient’s last visit.

The sponsor will notify the METC immediately of a temporary halt of the study, including the reason of such an action.

In case the study is ended prematurely, the sponsor will notify the accredited METC and the competent authority within 15 days, including the reasons for the premature termination.

Within one year after the end of the study, the investigator/sponsor will submit a final study report with the results of the study, including any publications/abstracts of the study, to the accredited METC and the Competent Authority.

12.6 Public disclosure and publication policy

Publication policy is in agreement with the CCMO publication statement. The results of the study will be published in peer-reviewed scientific journals. Both positive and negative results of the study will be disclosed. The principal investigator will publish and/or present results to the general public.

13. STRUCTURED RISK ANALYSIS

13.1 Potential issues of concern

a. Level of knowledge about mechanism of action

Pheburane is a FDA approved drug used for patients having urea cycle disorders. With the administration of Pheburane, its active substance Na-PB helps the body to get rid of excess nitrogen waste by bypassing the urea cycle (please see SPC text of Pheburane, document D2). Importantly, Na-PB activates also the BCKD complex, which can be seen as an 'off-target' effect. BCDK is the rate-limiting enzyme of the BCAA oxidation (see figure 5). With the activation of BCKD complex activity, BCAA oxidation will be enhanced thereby reducing BCAAs in plasma and muscle. In more detail, Na-PB inhibits the kinase regulating the BCKD complex activity via phosphorylation of the E1alpha subunit. The phosphorylation of the kinase will alleviate inhibition of the BCKD complex, thereby promoting BCAA oxidation [24, 39]. Together, activation of the BCKD complex by Na-PB in humans augments BCAA oxidation and lowers BCAA levels in plasma and muscle. Patients with T2D have elevated circulatory BCAA, possibly due lowered BCAA oxidation. This forms the rational to administer Pheburane in patients with T2D and to evaluate metabolic health.

Next to lowering BCAA, Na-PB is known to reduce ER stress and inhibit HDAC expression [49-52]. I am aware that the mechanistic interpretation of the results obtained from the intervention outlined in this project therefore could be somewhat complicated. However, the overall goal of the treatment of patients with T2D obviously is to promote insulin sensitivity. In that respect it is of relevance to note that 'off-target effects' like reduced ER stress and alterations in HDAC expression can be beneficial for patients with T2D as well. While taking the samples and during sample handling, we make sure to maintain the options for the exploration of 'off target parameters' like ER-stress and HDAC expression.

Thus, we will be in the position to explore the effects of Na-PB on insulin sensitivity and other key players determining metabolic health, with focus at the role of BCAA metabolism.

. b. Previous exposure of human beings with the test product(s) and/or products with a similar biological mechanism

Pheburane is a FDA approved drug. In this study Pheburane will be used as a tool to evaluate the role of BCAA metabolism on metabolic health in patients with T2D. The effect of Na-PB on BCAA metabolism has been carefully explored in (healthy) humans. More over, Na-PB has been successfully applied as a BCAA lowering agent, not only to enhance BCAA oxidation in MSUD patients characterized by a defective BCAA-oxidizing capacity and elevated circulating BCAA [53], but also in healthy humans possessing normal BCAA levels

before Na-PB was administered [24-28]. More specifically, in healthy people Na-PB induced a drop in leucine ranging from 26-51%, a drop in isoleucine between 33-50% and a drop in valine round 30%. Importantly, these studies performed in healthy humans having normal systemic BCAA to start with, administration with Na-PB did not caused any negative nitrogen balance or were in need for amino acid supplementation.

c. Can the primary or secondary mechanism be induced in animals and/or in ex-vivo human cell material?

Previously, I explored the mode of action of sodium phenylbutyrate in vitro in cultured human myocytes (figure 2, *data to be published*). Mitochondrial function increased in a doses-dependent manner. These ‘proof-of-concept’ pilot data obtained indicating that -in line with my hypothesis- muscle mitochondrial function improved upon administration of Na-PB to the petri-dish. However, with this project we will investigate whether these results can be obtained in vivo and explore if metabolic health will improve, of which the latter is not possible in the petri dish. Also, animal studies with the administration of Na-PB pronounced effects were found on elevating energy metabolism and on the lowering of circulatory BCAA [33, 54]. These results strengthen the working hypothesis outlined in this project.

d. Selectivity of the mechanism to target tissue in animals and/or human beings

With the administration of Na-PB, several target tissues and pathway become activated.

BCKD activity

Pages 14 and 15 contain detailed explanation on the expected effects of Na-PB exerted in muscle and liver. In humans, 50-60% of the oxidative capacity of BCKD resides within skeletal muscle. These data originate from the sole study that used molecular probes to measure the BCAA-catabolism by measuring the activity of BCKD and systematically compare the activity of a variety of tissues in man [20].

Insulin signaling pathway

Elevated levels of a mixture of amino acids were previously reported to directly impede with the insulin-stimulated glucose uptake in human muscle, causing insulin resistance via activation of signalling via mTOR and S6K [30, 31]. Therefore, in patients with T2D elevated circulating BCAA could activate mTOR and S6K thereby reducing insulin sensitivity. This has not been investigated yet.

Lipogenic genes

From animal studies it has been shown that elevated concentrations of BCAA may effect expression levels of lipogenic genes, resulting in higher fat storage [33, 34]. Together with my observation of the strong relation between BCAA and in vivo values for liver fat content (**figure 1b**), this supports the notion that BCAA are involved in fat depositioning in the liver.

Mitochondrial function

Hypothetically, low oxidation of BCAA could hamper the delivery of succinyl-CoA and acetyl-CoA into the TCA cycle, causing anaplerotic stress. Anaplerotic stress dysregulates substrate competition and results in reduction in TCA cycle capacity relative to fuel delivery [22, 23]. In this way, as depicted in **figure 2**, low BCAA oxidation underlie lower mitochondrial oxidative capacity. This is furthermore supported by the strong negative association I found between systemic BCAA and mitochondrial oxidative capacity as depicted in **figure 1c**. To summarize, low availability of the BCAA-derived intermediates due to low BCAA oxidation affect mitochondrial fuel flexibility and possibly the physiological insulin resistant state.

e. Analysis of potential effect

Moreover, it is of relevance to note that 75-80% of post-prandial glucose uptake in humans resides within skeletal muscle [29]. Hence, also modest improvements in BCKD activity in skeletal muscle percentage wise may have profound effects on whole body glucose uptake. In this study a maximal dosage of 4.8 g/m^2/day, which is below the lowest dose of clinical prescription.

The potential risks of Pheburane are described at page 27, paragraph 6.4. In short:

- a negative nitrogen balance within a short treatment period of 2 weeks, is not to be expected, however will be monitored during visits 2 (5) and 3 (6).
- adverse reactions like loss of appetite and changed body odor: this can be caused by phenylacetate and reduced temporary taste perception has been described for 3-4% of all patients with prolonged prescription. These reactions could compromise compliance, therefore, a dropout of ~20% is anticipated.
- amino acid deficiency: participants will be advised to keep their normal dietary habits, to exclude this risk. Levels of arginine and other (essential) amino acids will be monitored during visits 2 (5) and 3 (6).

f. Pharmacokinetic considerations

For details on pharmacokinetics, please find the SPC text for Pheburane. Also, see page 25 of this protocol.

g. Study population

Patients diagnosed with T2D. Patients with T2D are characterized by elevated circulatory BCAA. Na-BP will elevate These patients are relatively well-controlled with a HbA1c < 8.5% and without having co-morbidities. The responsible medical doctor assigned to this study

will carefully check inclusion and exclusion criteria. Also, the medical doctor knows the study drug from clinical practice.

h. Interaction with other products

Concurrent administration of probenecid may affect renal excretion of the conjugation product of sodium phenylbutyrate. There have been published reports of hyperammonemia being induced by haloperidol and by valproate. Corticosteroids may cause the breakdown of body protein and thus increase plasma ammonia levels. More frequent monitoring of plasma ammonia levels is advised when these medicinal products to be used

i. Predictability of effect

Appearance of side effects cannot be predicted as the used dosages are below the clinical prescription.

j. Can effects be managed?

The participants receive a card with the relevant information on the RCT a physician needs to know in case of emergency. In case of an emergency (related or not to the study medication), participants are advised to go to the emergency unit or general practitioner. In case of emergency the physician can contact the MUMC+ hospital pharmacy who has excess to the randomization codes for un-blinding for each individual participant. The randomization study code will be provided at the information card (see document F3.). The medication containers are also provided with the study code (NTR7426; EudraCT: 2018-003176-13). In case of emergency, the MUMC+ hospital pharmacy (Rogier van der Zanden), but as well the responsible medical physician (Dr. T. van de Weijer), the principle investigator (Dr. E. Phielix) and the secretary of the department of Nutrition and Movement Sciences (Yolanda Verhagen) can decipher whether study drug or placebo is administered. In less emergent situations, during working hours the researcher (Froukje Vanweert) or the PI (Dr. Esther Phielix) can be contacted. In any case, the responsible medical doctor (Dr. T. van de Weijer) will be contacted who will decide if the participant needs to discontinue the RCT.

13.2 Synthesis

Pheburane is a FDA-approved drug with well-described side effects or risk (see SPC text Pheburane and paragraph 6.4 and 13.1.e). In several studies, Na-PB has been administered in healthy humans and in people diagnosed with a disease (MSUD, urea cycle disorders, thalassemia) [24-28, 40]. No additional risks are expected with the administration of Pheburane in patients with T2D. Pheburane will be used as a tool to boost BCAA oxidation in patients with T2D.

14. REFERENCES

1. Phielix, E., et al., *Reduction of non-esterified fatty acids improves insulin sensitivity and lowers oxidative stress, but fails to restore oxidative capacity in type 2 diabetes: a randomised clinical trial.* Diabetologia, 2014. **57**(3): p. 572-81.
2. Phielix, E., et al., *Exercise training increases mitochondrial content and ex vivo mitochondrial function similarly in patients with type 2 diabetes and in control individuals.* Diabetologia, 2010. **53**(8): p. 1714-1721.
3. Phielix, E., et al., *Lower intrinsic ADP-stimulated mitochondrial respiration underlies in vivo mitochondrial dysfunction in muscle of male type 2 diabetic patients.* Diabetes, 2008. **57**(11): p. 2943-2949.
4. Szendroedi, J., E. Phielix, and M. Roden, *The role of mitochondria in insulin resistance and type 2 diabetes mellitus.* Nat Rev Endocrinol, 2012. **8**(2): p. 92-103.
5. Floegel, A., et al., *Identification of serum metabolites associated with risk of type 2 diabetes using a targeted metabolomic approach.* Diabetes, 2013. **62**(2): p. 639-48.
6. Newgard, C.B., et al., *A branched-chain amino acid-related metabolic signature that differentiates obese and lean humans and contributes to insulin resistance.* Cell Metab, 2009. **9**(4): p. 311-26.
7. Wang, T.J., et al., *Metabolite profiles and the risk of developing diabetes.* Nat Med, 2011. **17**(4): p. 448-53.
8. Felig, P., E. Marliss, and G.F. Cahill, Jr., *Plasma amino acid levels and insulin secretion in obesity.* N Engl J Med, 1969. **281**(15): p. 811-6.
9. Berger, M., et al., *Blood amine acid levels in patients with insulin excess (functioning insulinoma) and insulin deficiency (diabetic ketosis).* Metabolism, 1978. **27**(7): p. 793-9.
10. Glass, A.R., et al., *Normal valine disposal in obese subjects with impaired glucose disposal: evidence for selective insulin resistance.* Metabolism, 1981. **30**(6): p. 578-82.
11. Tai, E.S., et al., *Insulin resistance is associated with a metabolic profile of altered protein metabolism in Chinese and Asian-Indian men.* Diabetologia, 2010. **53**(4): p. 757-67.
12. Huffman, K.M., et al., *Relationships Between Circulating Metabolic Intermediates and Insulin Action in Overweight to Obese, Inactive Men and Women.* Diabetes Care, 2009. **32**(9): p. 1678-1683.
13. Palmer, N.D., et al., *Metabolomic profile associated with insulin resistance and conversion to diabetes in the Insulin Resistance Atherosclerosis Study.* J Clin Endocrinol Metab, 2015. **100**(3): p. E463-8.
14. Perng, W., et al., *Metabolomic profiles and childhood obesity.* Obesity (Silver Spring), 2014. **22**(12): p. 2570-8.
15. Shah, S.H., et al., *Branched-chain amino acid levels are associated with improvement in insulin resistance with weight loss.* Diabetologia, 2012. **55**(2): p. 321-30.
16. Walford, G.A., et al., *Metabolite Profiles of Diabetes Incidence and Intervention Response in the Diabetes Prevention Program.* Diabetes, 2016. **65**(5): p. 1424-33.
17. Pereira, S., et al., *Insulin resistance of protein metabolism in type 2 diabetes.* Diabetes, 2008. **57**(1): p. 56-63.
18. Lotta, L.A., et al., *Genetic Predisposition to an Impaired Metabolism of the Branched-Chain Amino Acids and Risk of Type 2 Diabetes: A Mendelian Randomisation Analysis.* PLoS Med, 2016. **13**(11): p. e1002179.
19. Mahendran, Y., et al., *Genetic evidence of a causal effect of insulin resistance on branched-chain amino acid levels.* Diabetologia, 2017. **60**(5): p. 873-878.
20. Suryawan, A., et al., *A molecular model of human branched-chain amino acid metabolism.* Am J Clin Nutr, 1998. **68**(1): p. 72-81.
21. Kornberg, H.L., P.J. Phizackerley, and J.R. Sadler, *The metabolism of C2 compounds in micro-organisms. 5. Biosynthesis of cell materials from acetate in Escherichia coli.* Biochem J, 1960. **77**: p. 438-45.
22. Muoio, D.M., *Metabolic inflexibility: when mitochondrial indecision leads to metabolic gridlock.* Cell, 2014. **159**(6): p. 1253-62.

23. Kelley, D.E. and L.J. Mandarino, *Fuel selection in human skeletal muscle in insulin resistance: a reexamination.* Diabetes, 2000. **49**(5): p. 677-83.
24. Brunetti-Pierri, N., et al., *Phenylbutyrate therapy for maple syrup urine disease.* Hum Mol Genet, 2011. **20**(4): p. 631-40.
25. Darmaun, D., et al., *Phenylbutyrate-induced glutamine depletion in humans: effect on leucine metabolism.* Am J Physiol, 1998. **274**(5 Pt 1): p. E801-7.
26. Le Bacquer, O., et al., *Acute depletion of plasma glutamine increases leucine oxidation in prednisone-treated humans.* Clin Nutr, 2007. **26**(2): p. 231-8.
27. Marini, J.C., et al., *Phenylbutyrate improves nitrogen disposal via an alternative pathway without eliciting an increase in protein breakdown and catabolism in control and ornithine transcarbamylase-deficient patients.* Am J Clin Nutr, 2011. **93**(6): p. 1248-54.
28. Scaglia, F., et al., *Effect of alternative pathway therapy on branched chain amino acid metabolism in urea cycle disorder patients.* Mol Genet Metab, 2004. **81 Suppl 1**: p. S79-85.
29. DeFronzo, R.A. and D. Tripathy, *Skeletal muscle insulin resistance is the primary defect in type 2 diabetes.* Diabetes Care, 2009. **32 Suppl 2**: p. S157-63.
30. Krebs, M., et al., *The Mammalian target of rapamycin pathway regulates nutrient-sensitive glucose uptake in man.* Diabetes, 2007. **56**(6): p. 1600-7.
31. Krebs, M., et al., *Mechanism of amino acid-induced skeletal muscle insulin resistance in humans.* Diabetes, 2002. **51**(3): p. 599-605.
32. Phielix, E. and M. Mensink, *Type 2 diabetes mellitus and skeletal muscle metabolic function.* Physiol Behav, 2008. **94**(2): p. 252-8.
33. Du, Y., et al., *Isoleucine or valine deprivation stimulates fat loss via increasing energy expenditure and regulating lipid metabolism in WAT.* Amino Acids, 2012. **43**(2): p. 725-34.
34. Cheng, Y., et al., *Leucine deprivation decreases fat mass by stimulation of lipolysis in white adipose tissue and upregulation of uncoupling protein 1 (UCP1) in brown adipose tissue.* Diabetes, 2010. **59**(1): p. 17-25.
35. Phielix, E., et al., *Lower intrinsic ADP-stimulated mitochondrial respiration underlies in vivo mitochondrial dysfunction in muscle of male type 2 diabetic patients.* Diabetes, 2008. **57**(11): p. 2943-9.
36. Capaldo, B., et al., *Carnitine improves peripheral glucose disposal in non-insulin-dependent diabetic patients.* Diabetes Res Clin Pract, 1991. **14**(3): p. 191-5.
37. Mingrone, G., et al., *L-carnitine improves glucose disposal in type 2 diabetic patients.* J Am Coll Nutr, 1999. **18**(1): p. 77-82.
38. Burrage, L.C., et al., *Sodium phenylbutyrate decreases plasma branched-chain amino acids in patients with urea cycle disorders.* Mol Genet Metab, 2014. **113**(1-2): p. 131-5.
39. Holecek, M., M. Vodenicarovova, and P. Siman, *Acute effects of phenylbutyrate on glutamine, branched-chain amino acid and protein metabolism in skeletal muscles of rats.* Int J Exp Pathol, 2017. **98**(3): p. 127-33.
40. Collins, A.F., et al., *Oral sodium phenylbutyrate therapy in homozygous beta thalassemia: a clinical trial.* Blood, 1995. **85**(1): p. 43-9.
41. Halvatsiotis, P., et al., *Synthesis rate of muscle proteins, muscle functions, and amino acid kinetics in type 2 diabetes.* Diabetes, 2002. **51**(8): p. 2395-404.
42. Luzi, L., A.S. Petrides, and R.A. De Fronzo, *Different sensitivity of glucose and amino acid metabolism to insulin in NIDDM.* Diabetes, 1993. **42**(12): p. 1868-77.
43. Welle, S. and K.S. Nair, *Failure of glyburide and insulin treatment to decrease leucine flux in obese type II diabetic patients.* Int J Obes, 1990. **14**(8): p. 701-10.
44. Bergstrom, J., et al., *Diet, muscle glycogen and physical performance.* Acta Physiol Scand, 1967. **71**(2): p. 140-50.
45. DeFronzo, R.A., J.D. Tobin, and R. Andres, *Glucose clamp technique: a method for quantifying insulin secretion and resistance.* American Journal of Physiology-Endocrinology And Metabolism, 1979. **237**(3): p. E214.
46. Royle, G.T., R.R. Wolfe, and J.F. Burke, *The measurement of glucose turnover and oxidation using radioactive and stable isotopes.* J Surg Res, 1983. **34**(2): p. 187-93.

47. Steele, R., *Influences of glucose loading and of injected insulin on hepatic glucose output.* Ann N Y Acad Sci, 1959. **82**: p. 420-30.
48. *World Medical Association Declaration of Helsinki: ethical principles for medical research involving human subjects.* Jama, 2013. **310**(20): p. 2191-4.
49. Cuadrado-Tejedor, M., et al., *Phenylbutyrate is a multifaceted drug that exerts neuroprotective effects and reverses the Alzheimer s disease-like phenotype of a commonly used mouse model.* Curr Pharm Des, 2013. **19**(28): p. 5076-84.
50. Tanis, R.M., et al., *The effect of glucose concentration and sodium phenylbutyrate treatment on mitochondrial bioenergetics and ER stress in 3T3-L1 adipocytes.* Biochim Biophys Acta, 2015. **1853**(1): p. 213-21.
51. Cho, J.A., et al., *4-Phenylbutyrate attenuates the ER stress response and cyclic AMP accumulation in DYT1 dystonia cell models.* PLoS One, 2014. **9**(11): p. e110086.
52. Asklund, T., et al., *Synergistic killing of glioblastoma stem-like cells by bortezomib and HDAC inhibitors.* Anticancer Res, 2012. **32**(7): p. 2407-13.
53. Kose, M., et al., *A Patient with MSUD: Acute Management with Sodium Phenylacetate/Sodium Benzoate and Sodium Phenylbutyrate.* Case Rep Pediatr, 2017. **2017**: p. 1045031.
54. da-Silva, W.S., et al., *The chemical chaperones tauroursodeoxycholic and 4-phenylbutyric acid accelerate thyroid hormone activation and energy expenditure.* FEBS Lett, 2011. **585**(3): p. 539-44.

CONSORT 2010 checklist of information to include when reporting a randomised trial*

Section/Topic	Item No	Checklist item	Reported on page No
**Title and abstract**			
	1a	Identification as a randomised trial in the title	1
	1b	Structured summary of trial design, methods, results, and conclusions (for specific guidance see CONSORT for abstracts)	3
**Introduction**			
Background and objectives	2a	Scientific background and explanation of rationale	5
	2b	Specific objectives or hypotheses	5
**Methods**			
Trial design	3a	Description of trial design (such as parallel, factorial) including allocation ratio	6
	3b	Important changes to methods after trial commencement (such as eligibility criteria), with reasons	NA
Participants	4a	Eligibility criteria for participants	6
	4b	Settings and locations where the data were collected	6
Interventions	5	The interventions for each group with sufficient details to allow replication, including how and when they were actually administered	6
Outcomes	6a	Completely defined pre-specified primary and secondary outcome measures, including how and when they were assessed	8
	6b	Any changes to trial outcomes after the trial commenced, with reasons	NA
Sample size	7a	How sample size was determined	8-9
	7b	When applicable, explanation of any interim analyses and stopping guidelines	NA
Randomisation:			
Sequence generation	8a	Method used to generate the random allocation sequence	6
	8b	Type of randomisation; details of any restriction (such as blocking and block size)	6
Allocation concealment mechanism	9	Mechanism used to implement the random allocation sequence (such as sequentially numbered containers), describing any steps taken to conceal the sequence until interventions were assigned	6
Implementation	10	Who generated the random allocation sequence, who enrolled participants, and who assigned participants to interventions	6
Blinding	11a	If done, who was blinded after assignment to interventions (for example, participants, care providers, those	6

		assessing outcomes) and how	
	11b	If relevant, description of the similarity of interventions	6
Statistical methods	12a	Statistical methods used to compare groups for primary and secondary outcomes	9
	12b	Methods for additional analyses, such as subgroup analyses and adjusted analyses	NA
**Results**			
Participant flow (a diagram is strongly recommended)	13a	For each group, the numbers of participants who were randomly assigned, received intended treatment, and were analysed for the primary outcome	10
	13b	For each group, losses and exclusions after randomisation, together with reasons	10
Recruitment	14a	Dates defining the periods of recruitment and follow-up	10
	14b	Why the trial ended or was stopped	
Baseline data	15	A table showing baseline demographic and clinical characteristics for each group	18
Numbers analysed	16	For each group, number of participants (denominator) included in each analysis and whether the analysis was by original assigned groups	23
Outcomes and estimation	17a	For each primary and secondary outcome, results for each group, and the estimated effect size and its precision (such as 95% confidence interval)	10 - 11
	17b	For binary outcomes, presentation of both absolute and relative effect sizes is recommended	NA
Ancillary analyses	18	Results of any other analyses performed, including subgroup analyses and adjusted analyses, distinguishing pre-specified from exploratory	NA
Harms	19	All important harms or unintended effects in each group (for specific guidance see CONSORT for harms)	10 - 11
**Discussion**			
Limitations	20	Trial limitations, addressing sources of potential bias, imprecision, and, if relevant, multiplicity of analyses	11-13
Generalisability	21	Generalisability (external validity, applicability) of the trial findings	11-13
Interpretation	22	Interpretation consistent with results, balancing benefits and harms, and considering other relevant evidence	11-13
**Other information**			
Registration	23	Registration number and name of trial registry	3
Protocol	24	Where the full trial protocol can be accessed, if available	
Funding	25	Sources of funding and other support (such as supply of drugs), role of funders	3 and 13

*We strongly recommend reading this statement in conjunction with the CONSORT 2010 Explanation and Elaboration for important clarifications on all the items. If relevant, we also recommend reading CONSORT extensions for cluster randomised trials, non-inferiority and equivalence trials, non-pharmacological treatments, herbal interventions, and pragmatic trials. Additional extensions are forthcoming: for those and for up to date references relevant to this checklist, see www.consort-statement.org.
