# Supplementary material for: A randomized placebo-controlled clinical trial for pharmacological activation of BCAA catabolism in patients with type 2 diabetes
Source: Nat Commun. 2022 Jun 18;13:3508. doi: 10.1038/s41467-022-31249-9 (PMC9206682; doi:10.1038/s41467-022-31249-9)
Supplement: Supplementary file 3 — Reporting Summary [file 41467_2022_31249_MOESM3_ESM.pdf]

nature portfolio

Corresponding author(s): Esther Phielix

Last updated by author(s): May 30, 2022

Reporting Summary

Nature Portfolio wishes to improve the reproducibility of the work that we publish. This form provides structure for consistency and transparency in reporting. For further information on Nature Portfolio policies, see our Editorial Policies and the Editorial Policy Checklist.

Statistics

For all statistical analyses, confirm that the following items are present in the figure legend, table legend, main text, or Methods section.

n/a	Confirmed	
☐	☒	The exact sample size (*n*) for each experimental group/condition, given as a discrete number and unit of measurement
☐	☒	A statement on whether measurements were taken from distinct samples or whether the same sample was measured repeatedly
☐	☒	The statistical test(s) used AND whether they are one- or two-sided<br>*Only common tests should be described solely by name; describe more complex techniques in the Methods section.*
☐	☒	A description of all covariates tested
☐	☒	A description of any assumptions or corrections, such as tests of normality and adjustment for multiple comparisons
☐	☒	A full description of the statistical parameters including central tendency (e.g. means) or other basic estimates (e.g. regression coefficient) AND variation (e.g. standard deviation) or associated estimates of uncertainty (e.g. confidence intervals)
☐	☒	For null hypothesis testing, the test statistic (e.g. *F*, *t*, *r*) with confidence intervals, effect sizes, degrees of freedom and *P* value noted<br>*Give P values as exact values whenever suitable.*
☒	☐	For Bayesian analysis, information on the choice of priors and Markov chain Monte Carlo settings
☒	☐	For hierarchical and complex designs, identification of the appropriate level for tests and full reporting of outcomes
☐	☒	Estimates of effect sizes (e.g. Cohen's *d*, Pearson's *r*), indicating how they were calculated

Our web collection on statistics for biologists contains articles on many of the points above.

Software and code

Policy information about availability of computer code

Data collection	No software was used
Data analysis	Results were analyzed using SPSS 26.0 for Mac

For manuscripts utilizing custom algorithms or software that are central to the research but not yet described in published literature, software must be made available to editors and reviewers. We strongly encourage code deposition in a community repository (e.g. GitHub). See the Nature Portfolio guidelines for submitting code & software for further information.

Data

Policy information about availability of data

All manuscripts must include a data availability statement. This statement should provide the following information, where applicable:

- Accession codes, unique identifiers, or web links for publicly available datasets
- A description of any restrictions on data availability
- For clinical datasets or third party data, please ensure that the statement adheres to our policy

The dataset generated during and analyzed during the current study are not publicly available. The corresponding author (E.P.) is the custodian of the data and will provide access to de-identified and processed participant data for academic purposes on request (esther.phielix@maastrichtuniversity.nl), with the completion of a data access agreement. The source data underlying Fig. 2 and 3, and supplementary Fig. 1 are provided as a Source Data file. Source data and study protocol are provided with this paper immediately following publication with no end date.

Field-specific reporting

Please select the one below that is the best fit for your research. If you are not sure, read the appropriate sections before making your selection.

☒ Life sciences ☐ Behavioural & social sciences ☐ Ecological, evolutionary & environmental sciences

For a reference copy of the document with all sections, see nature.com/documents/nr-reporting-summary-flat.pdf

Life sciences study design

All studies must disclose on these points even when the disclosure is negative.

Sample size	We calculated (G*Power 3.1 software, Faul, Erdfelder, Land and Buchner, University of Trier) that 15 participants were needed to complete the study to achieve an effect size of 0.93, power of 0.90 and a significance level of 0.05 (two-sided). Accounting for a possible drop-out rate of 20%, a minimal of 18 participants needed to be randomized into the study.
Data exclusions	Fasting glucose NaPB01: protocol deviation in NaPB condition<br>Fasted FFA, Insulin and TG NaPB08: vagus reflex during biopsy clamp<br>Glucose during clamp NaPB08: vagus reflex during biopsy clamp<br>Insulin and FFA during clamp NaPB08 excluded from analysis: vagus reflex during biopsy<br>Substrate oxidation during clamp NaPB08 and NaPB13: bad quality indirect calorimetry<br>Rd, EGP and NOGD clamp:<br>o NaPB08: vagus reflex during biopsy clamp<br>o NaPB16: incorrect values tracer data<br>o NaPB13 (NOGD) bad quality indirect calorimetry<br>Liver fat content and composition:<br>o Fat conten NaPB02 andNaPB14: bad quality MRS spectra<br>o Composition NaPB02, NaPB08 and NaPB16: bad quality MRS spectra
Replication	No measurements were replicated due to invasiveness of the study.
Randomization	Random allocation to NaPB or placebo was done by an independent researcher of Maastricht University using an interactive online response system that randomly allocated the participants to on of the two intervention groups using controlled randomization. Envelops were available containing the unblinding key for both intervention arms.
Blinding	We used a double-blind design in which patients received the active drug or placebo orally. The active products and corresponding placebo products were visually identical to maintains masking of patients and researchers. Researchers were masked at the end of the trial to analyze the data.

Reporting for specific materials, systems and methods

We require information from authors about some types of materials, experimental systems and methods used in many studies. Here, indicate whether each material, system or method listed is relevant to your study. If you are not sure if a list item applies to your research, read the appropriate section before selecting a response.

Materials & experimental systems

n/a	Involved in the study	
☒	☐	Antibodies
☒	☐	Eukaryotic cell lines
☒	☐	Palaeontology and archaeology
☒	☐	Animals and other organisms
☐	☒	Human research participants
☐	☒	Clinical data
☒	☐	Dual use research of concern

Methods

n/a	Involved in the study	
☒	☐	ChIP-seq
☒	☐	Flow cytometry
☒	☐	MRI-based neuroimaging

Human research participants

Policy information about studies involving human research participants

Population characteristics	Thirteen male and 3 postmenopausal females diagnosed with type 2 diabetes for at least 1.5 years with a mean age of 66 years treated with oral glucose lowerming medication (metformin only, or in combination with sulphonylurea agents and/or DPPIV inhibitors)
Recruitment	Subjects were recruited in Maastricht and surroundings by means of posters, flyers and advertisements in local newspapers and on the internet.

	Participants from previous studies were contacted as well in case they provided written consent for this. To increase recruitment efficiency, potential participants could register at a mailing list. With this list, the researcher could approach individuals who showed interest in participating scientific research.<br>The researchers contacted individuals who were interested in participating in the study by telephone, only after the individual had sought contact with the researcher on his or her accord first. By contacting responders by telephone first, the burden of travel and time effort was reduced for the potential subject and researchers. In the telephone interview, the goal of the study was explained, and the basic inclusion criteria was discussed. When responders were interested, they received detailed subject information via e-mail or mail accompanied by a general brochure (provided by the Dutch government) about participating in a medical study. They were instructed to read this information carefully and to ask questions if things were unclear. The researcher contacted the possible participant again at least 7 days after the study information was received by the participant. If responders wanted to participate after reading the study information and seemed to be eligible, they were invited for a screening.<br>The subjects received a compensation for their contribution to the study. After complete participations, participants received 800 euros.
Ethics oversight	Medical Review Ethics Committee of Maastricht University and Medical Centre

Note that full information on the approval of the study protocol must also be provided in the manuscript.

Clinical data

Policy information about clinical studies

All manuscripts should comply with the ICMJE guidelines for publication of clinical research and a completed CONSORT checklist must be included with all submissions.

Clinical trial registration	Netherlands Trial Register ID: NTR7426)
Study protocol	The protocol can be found on the website of Netherlands Trial Register: https://www.trialregister.nl/trial/7227
Data collection	Data was collected between February 2019 and February 2020 in Maastricht University Medical Center, the Netherlands.
Outcomes	The primary outcome was peripheral insulin sensitivity, measured by the hyperinsulinemic-euglycemic clamps, expressed as the change in insulin-stimulated glucose disposal rate minus baseline (ΔRd).<br>Secondary outcomes were ex vivo mitochondrial oxidative capacity in skeletal muscle, measured with high-resolution respirometry expressed as O2-flux, substrate oxidation, assessed with indirect calorimetry and ectopic fat accumulation in muscle and liver measured with proton magnetic resonance spectroscopy (1H-MRS). Fasting blood samples were collected to determine levels of BCAA and their intermediates and glucose. In addition, phenylbutyrate levels were determined by LCMS to check compliance to the intervention.
